# Cholesterol- and Biosurfactant-Stabilized Ethosomes for Betanin: Formulation, Encapsulation and Controlled In Vitro Release

**DOI:** 10.3390/ijms27219548

**Published:** 2026-10-26

**Authors:** Jagoda Chudzińska-Skorupinska, Agata Wawrzyńczak, Agnieszka Feliczak-Guzik

**Affiliations:** Department of Applied Chemistry, Faculty of Chemistry, Adam Mickiewicz University in Poznań, Uniwersytetu Poznańskiego 8, 61-614 Poznań, Poland; jagoda.chudzinska@amu.edu.pl

**Keywords:** ethosomes, biosurfactant, cholesterol, encapsulation, betanin, controlled release

## Abstract

Betanin is a hydrophilic antioxidant with limited physicochemical stability, motivating the development of vesicular carriers to improve its retention and regulate release. This study employed a systematic, stepwise formulation-screening approach to develop and characterize cholesterol-containing ethosomes, including biosurfactant-modified formulations, for betanin encapsulation. The effects of preparation conditions, surfactant type and concentration, cholesterol content, and betanin loading on colloidal properties and storage stability were evaluated. Encapsulation efficiency, loading capacity, and in vitro release were also investigated. Formulations containing 2.5% (*w*/*w*) phospholipid, 30% (*w*/*w*) ethanol, 10% (*w*/*w*) ethylene glycol, 1% (*w*/*w*) cholesterol, and 0.625% (*w*/*w*) Tween 20 or Tween 20/Mirasoft^®^ SL L60 mixture, prepared by 3 min homogenization, showed the most favorable performance. Betanin incorporation altered vesicle organization, while encapsulation and release strongly depended on surfactant composition and betanin loading. The highest encapsulation efficiency was 87.87 ± 3.22%, whereas the maximum loading capacity was 0.0271 ± 0.0026%. Release was incomplete and composition-dependent, ranging from 5.67 to 34.49% after 90 min at pH 5.8. The limited release indicated formulation-dependent betanin retention, which may favor controlled delivery but could also restrict active-compound availability. As skin permeation and biological safety were not evaluated, the relevance of these findings for dermal delivery remains to be established. Weibull analysis adequately described the nonlinear release profiles. These findings demonstrate that biosurfactant-modified ethosomes are tunable lipid nanocarriers with composition-dependent encapsulation and in vitro release properties.

## 1. Introduction

Transdermal drug delivery systems represent an attractive alternative to conventional routes of administration because they may reduce the invasiveness of therapy, improve patient comfort, and provide controlled and prolonged release of active compounds [1,2]. Despite their numerous advantages, their effectiveness remains limited by the stratum corneum, which constitutes the main barrier to the transport of both hydrophilic and lipophilic molecules [1,3,4]. Therefore, the development of advanced drug delivery systems (DDS) capable of increasing compound bioavailability, while maintaining formulation stability and a controlled release profile, remains one of the major directions of contemporary research [5]. We can identify several types of lipid-based vesicular carriers, a comparison of which is provided in Table 1.

Among lipid-based nanocarriers, ethosomes have attracted particular interest. These flexible vesicles are composed of phospholipids, ethanol, and water. The high ethanol content increases the fluidity and deformability of the lipid membrane and simultaneously interacts with the lipids of the stratum corneum, thereby facilitating the transport of encapsulated active compounds [16,17]. Owing to their greater deformability compared with conventional liposomes, ethosomes are regarded as among the most promising carriers for the topical and transdermal delivery of bioactive compounds, including naturally derived substances [18]. However, the effectiveness of ethosomal delivery systems depends on numerous interrelated formulation and technological factors. Surfactant type and concentration, ethanol content, the presence of cholesterol, and homogenization parameters affect the hydrodynamic diameter (D_h_), polydispersity index (PDI), zeta potential (ZP), vesicle morphology, encapsulation efficiency, and release profile of the active compound [8]. Cholesterol plays a particularly important role by increasing lipid membrane organization, reducing membrane permeability, and limiting leakage of the encapsulated compound. However, excessive membrane stabilization may decrease vesicle flexibility, which is considered one of the most important properties determining the effectiveness of ethosomes in transdermal delivery. Optimization of the cholesterol content therefore requires a balance among formulation stability, encapsulation efficiency, and controlled release of the active compound [19,20]. Biosurfactants are also attracting increasing interest as more biocompatible and sustainable alternatives to conventional synthetic surfactants. Their presence may affect lipid membrane organization, surface properties, and the colloidal stability of ethosomes [18]. The simultaneous use of a synthetic surfactant and a biosurfactant offers the possibility of tailoring the properties of the system while reducing the total content of surface-active compounds.

Natural antioxidants are currently being extensively investigated because of their ability to reduce oxidative stress and the associated inflammatory processes. One of the best-characterized representatives of this group is betanin, the principal betalain pigment in beetroot and a major contributor to the biological activity of this plant material [21,22]. Despite its high biological potential, the practical application of betanin remains limited because of its hydrophilic nature, low physicochemical stability, and susceptibility to degradation under the influence of environmental factors. Betanin, therefore, represents a challenging yet valuable model compound for studies on modern controlled delivery systems. The antioxidant activity of natural compounds may be evaluated using methods based on hydrogen atom transfer (HAT) and single-electron transfer (SET) mechanisms [23,24,25,26]. The most commonly used methods include the DPPH [27,28], ABTS [29,30], CUPRAC [31,32,33,34], and FRAP [35,36,37] assays, as well as the Folin–Ciocalteu method [38,39,40]. Because each of these methods is based on a different reaction mechanism, their combined application enables a comprehensive assessment of the antioxidant properties of the samples. Beetroot is one of the richest natural sources of betalains and exhibits high antioxidant activity, as confirmed, among others, by the DPPH assay. Its activity exceeds that of many commonly consumed fruits and vegetables, such as tomatoes and oranges [41]. These properties further support the selection of betanin as a model active compound in studies on ethosomal delivery systems.

The uniqueness of betanin’s application in this study comes from combining this natural dye with an ethosomal system of modified composition. Betanin is a compound with high functional value, but its use is limited by susceptibility to degradation under the influence of, for example, temperature, light, and oxygen. For this reason, the development of a suitable delivery system is crucial for increasing betanin’s stability and practical applicability. Previous studies on betanin encapsulation have involved the use of various carrier systems [42], including liposomes [43,44,45], chitosan-surface-modified liposomes [46,47,48], multiple emulsions [49], systems based on maltodextrin and gum arabic [50], maltodextrin and inulin [51], pullulan [52], and alginate [53]. In 2026, the report was also published on the simultaneous encapsulation of betanin and curcumin in an ethosome-based gel system [54].

Despite the large number of studies devoted to ethosomes, comprehensive analyses simultaneously assessing the effects of cholesterol content, surfactant type, biosurfactant incorporation, and homogenization parameters on physicochemical properties, stability, encapsulation efficiency, and the release profile of the active compound are still lacking. Reports on ethosomal formulations intended for the encapsulation of natural antioxidants, particularly betanin, also remain scarce. Addressing this knowledge gap may provide valuable information supporting the rational design of stable and effective lipid-based delivery systems. According to the available literature, the use of ethosomes modified with cholesterol and a surfactant mixture containing Mirasoft^®^ SL L60 and Tween 20 as a delivery system for betanin has, to the best of our knowledge, not yet been described. The proposed formulation aims not only to encapsulate betanin but, above all, to systematically modulate the properties of the vesicle membrane and the interfacial region, which may enhance the integrity of the system, limit aggregation and leakage of the active substance, and improve its stability during storage. Thus, the system designed in this way represents a potentially valuable approach to protecting betanin from degradation and increasing its retention within the carrier, and its development and characterization contribute to further research on delivery systems for this pigment.

The potential advantages of the developed system stem from the complementary roles of phospholipids, cholesterol, Mirasoft^®^ SL L60, and Tween 20, which together influence the structural and interfacial properties of the ethosomes. Phospholipids form the basic structure of the vesicle membrane, while cholesterol increases its order, limits permeability, and may reduce the risk of leakage of the active compound and vesicle fusion. Tween 20, as a nonionic surfactant, modifies the membrane properties and can influence the size, flexibility, and transport properties of the vesicles [55]. Mirasoft^®^ SL L60, a biosurfactant belonging to the sophorolipid group, may interact with the vesicle surface and contribute to steric stabilization, thereby potentially limiting aggregation and improving dispersion stability [8]. Appropriate selection of the proportions of these components can therefore reduce vesicle destabilization and betanin leakage, potentially limiting its direct exposure to environmental factors that promote degradation. Consequently, a more stable microenvironment for betanin may be provided, which could help maintain its stability during storage. The essence of this approach is thus the systematic modulation of the structural and interfacial properties of ethosomes through the combined selection of formulation components and process parameters, rather than the action of a single component as an isolated protective barrier.

Therefore, the aim of the present study was to develop and optimize ethosomal carrier systems containing betanin as a model natural bioactive compound. In the first stage, the antioxidant activity of beetroot extract and pure betanin was evaluated to support the selection of the active compound for encapsulation. Subsequently, a systematic analysis was conducted to determine the effects of cholesterol content, surfactant type, biosurfactant incorporation, and homogenization parameters on hydrodynamic diameter, polydispersity index, zeta potential, encapsulation efficiency, morphology, storage stability, and the in vitro release profile of betanin. Since skin penetration and retention studies were beyond the scope of the present work, the developed formulations should be regarded as potential platforms for the topical and transdermal delivery of betanin that require further biological evaluation.

## 2. Results and Discussion

### 2.1. Characterization of Beetroot Extract

Based on the HPLC-UV-Vis chromatograms of the beetroot extract and the betanin standard solution (Figure S1), together with the corresponding calibration curve (Figure S2), the betanin concentration in the extract was determined to be 7.56 mg/mL. This result justified the preparation of a 7.5 mg/mL betanin working solution, which was subsequently used as the model active compound for the modification of ethosomes.

### 2.2. Antioxidant Activity of the Beetroot Extract and Betanin

The antioxidant activity of the beetroot extract and betanin solution was evaluated using the DPPH, ABTS, CUPRAC, FRAP, and Folin–Ciocalteu assays. The application of several complementary methods enabled comparison of free-radical-scavenging activity with reducing capacity. The percentage inhibition values obtained in the DPPH and ABTS assays (Table S1) should be regarded as relative indicators because they depend on sample concentration, incubation time, and measurement conditions [56,57,58]. Therefore, quantitative comparisons were based primarily on the Trolox^®^ equivalent values. The Trolox^®^ calibration curves for the DPPH, ABTS, CUPRAC, FRAP, and Folin–Ciocalteu assays are presented in the Supplementary Materials (Figures S3–S7). In all cases, a linear relationship between the analytical response and Trolox^®^ concentration was observed over the range of 0.1–1.0 mmol/L, confirming the suitability of the selected calibration ranges. These curves were used to calculate the Trolox^®^ equivalent values (µmol TE/mL) for the beetroot extract and betanin solution.

The beetroot extract exhibited high activity in the DPPH and ABTS assays, reaching 165.9 and 148.7 µmol TE/mL, respectively, indicating its considerable free-radical-scavenging potential (Table 2). This finding is consistent with previous reports demonstrating the high activity of beetroot extracts in the DPPH and ABTS assays [51]. Betanin at a concentration of 7.5 mg/mL showed particularly high activity in the ABTS assay, reaching 226.5 µmol TE/mL. This result confirms its strong radical-scavenging properties and supports its use as the active compound in the subsequent formulation studies. Similar relationships have been reported for betacyanins, including betanin, which exhibit high activity in DPPH and ABTS assays but lower values when assessed using the FRAP method [59,60].

The lower Trolox^®^ equivalent values determined using the CUPRAC and FRAP assays compared with those obtained in the DPPH and ABTS assays indicate differences in the responses of the investigated samples to the applied chemical assays, reflecting their distinct reaction mechanisms and experimental conditions. In turn, the results of the Folin–Ciocalteu assay provide additional information on the reducing capacity of betanin. However, this method is not specific to phenolic compounds and does not allow the contribution of individual functional groups to be determined.

The results summarized in Table 2 confirm the substantial in vitro radical-scavenging and reducing capacity of betanin and support its selection as a model bioactive compound for encapsulation in ethosomes.

### 2.3. Effect of the Preparation Method on Ethosome Properties

In the first stage of the study, the effect of the preparation method on the physicochemical properties of ethosomes containing 1% (*w*/*w*) cholesterol and 1.25% (*w*/*w*) Tween 80 or Tween 20 was evaluated. Systems prepared using the cold method with stirring were compared with those subjected to homogenization for 3 or 6 min.

After 14 days of refrigerated storage, the Tween 80-containing samples prepared using stirring alone (C-yE-z8T-1) and 3 min homogenization (H-C-yE-z8T-1) exhibited comparable hydrodynamic diameters of approximately 220 d.nm. At the same time, their PDI values remained relatively high, amounting to 0.476 and 0.436, respectively, which indicates moderate dispersion homogeneity. Extending the homogenization time to 6 min did not improve either the hydrodynamic diameter or the PDI, but increased the absolute zeta potential to −49.73 mV for the yH-C-yE-z8T-1 sample (Table S2, Figure S8).

For the Tween 20-containing samples, a different relationship was observed. Homogenization reduced the hydrodynamic diameter compared with the non-homogenized sample. However, it did not consistently improve the polydispersity index or zeta potential after 14 days. The sample prepared using stirring alone (C-yE-z2T-1) exhibited a more negative zeta potential (−38.87 mV) than the homogenized samples, for which ZP values were approximately −25 mV. This indicates that increasing the intensity of homogenization did not directly improve all critical quality attributes of the ethosomes (Table S2, Figure S8).

The results indicate that prolonged homogenization was not beneficial as a universal method for improving system stability. Although it increased the absolute value of the zeta potential in some cases, it did not simultaneously reduce D_h_ and PDI. Therefore, 3 min homogenization was selected for the subsequent optimization stages, as it represented a compromise between effective size reduction, colloidal stability, and limitation of excessive mechanical energy that could promote vesicle reorganization or aggregation.

The addition of cholesterol improves the structural stability of vesicles, as reflected by lower polydispersity index values and smaller changes in hydrodynamic diameter during storage compared with cholesterol-free samples of the same composition [8]. By incorporating between phospholipid molecules, cholesterol increases the rigidity of the lipid bilayer, reduces its fluidity, and limits the tendency toward coalescence and structural reorganization. Consequently, cholesterol-containing systems exhibit greater long-term stability and higher homogeneity [61].

### 2.4. Effect of Surfactant Concentration on the Physicochemical Properties of Ethosomes

Reducing the surfactant concentration from 1.25% to 0.625% (*w*/*w*) decreased the PDI values in all evaluated samples, indicating improved uniformity of the particle-size distribution. For the Tween 80-containing formulations, the hydrodynamic diameters (D_h_) of samples with the lower and higher surfactant concentrations were comparable, whereas the most pronounced effect of surfactant reduction was observed for the Tween 20-containing systems. Decreasing the Tween 20 concentration to 0.625% (*w*/*w*) reduced the hydrodynamic diameter to 199.93 d.nm for H-C-yE-x2T-1 sample and to 291.97 d.nm for yH-C-yE-x2T-1 sample. At the same time, the zeta potential values after 14 days of storage remained comparable across all analyzed samples and ranged from −30.07 to −33.27 mV, indicating that favorable electrostatic stabilization of the systems was maintained (Table S3, Figure S9).

A particularly favorable effect was observed for the Tween 20-containing formulations, in which reducing the surfactant concentration simultaneously decreased the hydrodynamic diameter and improved system homogeneity. This effect should be regarded as formulation-dependent because the properties of ethosomal and transethosomal vesicular systems are determined not only by the presence of a surfactant but also by its type, concentration, and interactions with the phospholipid, ethanol, and other formulation components. The literature indicates that surfactants acting as edge activators can substantially modulate hydrodynamic diameter, PDI, zeta potential, colloidal stability, membrane flexibility, and the ability to penetrate the skin [62,63,64,65,66]. At the same time, excessive surfactant concentrations in formulations intended for dermal application may increase the risk of irritation. Therefore, the use of the lowest effective surfactant concentration is advantageous from the perspective of safety and rational formulation design [67].

### 2.5. Effect of Surfactant Type on the Physicochemical Properties of Ethosomes

The effect of surfactant type on the physicochemical properties of ethosomes was evaluated by comparing systems containing the individual surfactants Tween 20 or Tween 80 with systems containing mixtures of these surfactants and the Mirasoft^®^ SL L60 biosurfactant. After 14 days of storage, samples containing the Tween 20/Mirasoft^®^ SL L60 mixture (xMixY) exhibited similar zeta potential values of approximately −33 mV, irrespective of homogenization time (Table S4). This indicates that replacing Tween 20 alone with the surfactant mixture did not impair the electrostatic stabilization of the systems. At the same time, the use of the Tween 20/Mirasoft^®^ SL L60 mixture resulted in a pronounced decrease in hydrodynamic diameter compared with formulations containing Tween 20 alone. For the sample homogenized for 3 min, the hydrodynamic diameter decreased from 199.93 d.nm for H-C-yE-x2T-1 sample to 145.83 d.nm for H-C-yE-xMixY-1 sample. A similar, even more pronounced effect was observed for the samples homogenized for 6 min, for which D_h_ decreased from 291.97 d.nm for yH-C-yE-x2T-1 to 128.67 d.nm for yH-C-yE-xMixY-1. In addition, PDI values decreased in both cases, indicating improved homogeneity of the vesicle population (Figure S10).

A different effect was observed for systems containing the Tween 80/Mirasoft^®^ SL L60 mixture (xMix). Compared with formulations containing Tween 80 alone, use of the mixture reduced the absolute value of the zeta potential from approximately −30 mV to −28.40 mV for the sample homogenized for 3 min and to −24.17 mV for the sample homogenized for 6 min (Table S4). For the sample subjected to shorter homogenization, an increase in hydrodynamic diameter was observed together with a decrease in PDI, whereas extending homogenization to 6 min resulted in more than a twofold increase in both D_h_ and PDI. These results indicate that the effect of Mirasoft^®^ SL L60 depended on the type of surfactant included in the mixture and on the homogenization conditions (Figure S10).

The presence of cholesterol was also found to modify the properties of systems prepared using the same method, affecting both PDI and zeta potential values [9]. In samples containing the Tween 80-based mixture, the presence of cholesterol was associated with an increase in hydrodynamic diameter, whereas in samples containing the Tween 20/Mirasoft^®^ SL L60 mixture, a more than 3.5-fold reduction in D_h_ was observed. This suggests that the beneficial effect of the biosurfactant was most pronounced in Tween 20-containing systems, in which more efficient organization of the components at the interface and stabilization of smaller vesicles probably occurred.

These findings are consistent with the general role of surfactants, also referred to as edge activators in ultra-deformable systems, which can modulate vesicle flexibility, surface stability, and size. However, their effects depend not only on concentration but also on surfactant type and its interactions with phospholipids, cholesterol, and ethanol present within the lipid bilayer. In transethosomal systems, the presence of ethanol and surfactant increases vesicle deformability and may facilitate vesicle reorganization. However, an excessive or inadequately selected surfactant concentration may increase system heterogeneity [68].

Overall, the use of the Tween 20/Mirasoft^®^ SL L60 mixture resulted in more favorable colloidal properties than the use of Tween 20 alone, particularly with respect to hydrodynamic diameter and PDI. The most favorable properties were observed for formulations containing the Tween 20/Mirasoft^®^ SL L60 mixture and prepared using 3 min homogenization. These findings indicate that Mirasoft^®^ SL L60 did not merely serve as a naturally derived additive but acted as a component that modulated the interfacial properties, homogeneity, and stability of the ethosomal systems. The importance of surfactant type and its combination with other carrier components has also been demonstrated in studies on transethosomes, in which surfactant combinations influenced surface properties, application potential, and the safety of transdermal formulations [65].

### 2.6. Impact of Cholesterol Concentration on the Physicochemical Properties of Ethosomes

In the next stage of the study, the effect of reducing the cholesterol concentration from 1.0% to 0.5% (*w*/*w*) on the physicochemical properties of ethosomes containing Tween 20/Mirasoft^®^ SL L60 or Tween 80/Mirasoft^®^ SL L60 surfactant mixtures was evaluated. After 14 days of storage, reducing the cholesterol concentration increased the hydrodynamic diameter of samples containing the Tween 20/Mirasoft^®^ SL L60 mixture (xMixY), both for formulations prepared using 3 and 6 min of homogenization. At the same time, the effect of this change on PDI depended on the homogenization time. For the sample homogenized for 3 min, the PDI decreased from 0.209 to 0.126, whereas for its counterpart homogenized for 6 min, it increased from 0.240 to 0.304. The zeta potential values were less favorable for formulations containing 0.5% (*w*/*w*) cholesterol and, after 14 days, were −21.43 mV for H-xC-yE-xMixY-1 sample and −18.13 mV for yH-xC-yE-xMixY-1 sample (Table S5, Figure S11).

For formulations containing the Tween 80/Mirasoft^®^ SL L60 mixture (xMix), the effect of reducing the cholesterol concentration also depended on the homogenization conditions. For the sample prepared using 3 min of homogenization, reducing the cholesterol concentration increased both the hydrodynamic diameter and PDI, indicating deterioration of system homogeneity. In contrast, for the sample homogenized for 6 min, decreases in both D_h_ and PDI were observed. After 14 days, the zeta potential values of the formulations containing 0.5% *w*/*w*) cholesterol were −29.07 mV for H-xC-yE-xMix-1 sample and −23.33 mV for yH-xC-yE-xMix-1 sample, confirming that electrostatic stabilization depended on the combined effects of surfactant composition and homogenization energy (Table S5, Figure S11).

Overall, cholesterol affected ethosome stability. However, its effect was not uniform and depended on surfactant type and preparation conditions. The presence of 1.0% (*w*/*w*) cholesterol favored more advantageous zeta potential and PDI values in selected formulations, particularly in systems containing the Tween 20/Mirasoft^®^ SL L60 mixture. Reducing the cholesterol concentration to 0.5% (*w*/*w*) did not consistently improve the colloidal parameters and, in some cases, resulted in an increased hydrodynamic diameter or a decrease in the absolute zeta potential after 14 days of storage.

The literature indicates that cholesterol stabilizes the lipid bilayer by incorporating between phospholipid molecules, thereby limiting excessive membrane fluidity and reducing the tendency of vesicles to undergo coalescence and structural reorganization [61,69]. At the same time, its presence may reduce membrane flexibility. Therefore, the incorporation of cholesterol into ethosomes represents a compromise between structural stability and vesicle deformability [69]. Similar relationships have been reported for lipid-based vesicular systems in which cholesterol increased membrane ordering and reduced permeability, although the overall effect depended on the cholesterol-to-phospholipid ratio and the presence of additional surface-active components [70]. In the investigated systems, the higher cholesterol concentration of 1.0% (*w*/*w*) was considered more favorable for further formulation development because it provided a better balance among colloidal stability, homogeneity, and zeta potential.

### 2.7. Physicochemical Characterization of Betanin-Loaded Ethosomes

After 14 days of storage, samples containing the Tween 20/Mirasoft^®^ SL L60 surfactant mixture (xMixY) exhibited an increase in hydrodynamic diameter with increasing betanin solution concentration. The D_h_ value increased from 239.70 d.nm for the sample containing 1.0% (*w*/*w*) betanin solution to 416.63 d.nm for the sample containing 5.0% (*w*/*w*) betanin solution. A similar trend was observed for the absolute zeta potential values, which increased with increasing betanin content. The lowest PDI in this series was observed for the sample containing 3.0% (*w*/*w*) betanin solution, for which the PDI was 0.145, whereas the sample with the highest betanin concentration exhibited the highest PDI, equal to 0.315 (Table S6, Figure S12).

For samples containing Tween 20 as the sole surfactant (x2T), the formulations containing 1.0% and 3.0% (*w*/*w*) betanin solution exhibited comparable hydrodynamic diameters of approximately 340 d.nm. In contrast, the sample containing the highest betanin concentration was characterized by a smaller hydrodynamic diameter of 248.50 d.nm. The lowest PDI was also recorded for the sample containing 3.0% (*w*/*w*) betanin solution, for which the PDI was 0.261. The PDI of the sample containing 1.0% (*w*/*w*) betanin solution was 0.380, whereas the highest PDI, equal to 0.519, was observed for the sample with the highest betanin concentration. In this series, the increase in the absolute zeta potential value was particularly pronounced for the sample containing 5.0% (*w*/*w*) betanin solution, which reached −34.57 mV after 14 days (Table S6, Figure S12).

Overall, betanin incorporation affected the hydrodynamic diameter, PDI, and zeta potential of the ethosomes, indicating that the presence of the active compound modified vesicle organization. The lowest PDI values in both analyzed series were observed for formulations containing the intermediate betanin solution concentration of 3.0% (*w*/*w*), suggesting the existence of an optimal range of betanin content within the carrier. At the highest betanin concentration, an increase in PDI was observed, particularly in the system containing Tween 20 alone, which may indicate increased formulation heterogeneity at higher active-compound loading.

### 2.8. Statistical Analysis of Physicochemical Stability Data

Statistical analysis confirmed that both formulation composition and storage time significantly affected the physicochemical properties of the investigated ethosomal systems. A total of 123 omnibus tests were performed for zeta potential, hydrodynamic diameter, and PDI. Depending on the data distribution and homogeneity of variance, either one-way analysis of variance (ANOVA) or the Kruskal–Wallis test was applied. Statistically significant differences were identified in 117 of the 123 comparisons, confirming that formulation composition, preparation conditions, and storage time were important determinants of colloidal stability of ethosomes. A detailed summary of the statistical analysis is provided in the Supplementary Materials (Tables S7–S9).

### 2.9. Evaluation of Betanin Encapsulation Efficiency and Loading Capacity

Encapsulation efficiency (EE%) and loading capacity (LC%) are key parameters for evaluating vesicular systems because they describe two distinct aspects of active-compound incorporation into the carrier. EE% indicates the proportion of the initially added betanin that was retained within the formulation, whereas LC% refers to the amount of active compound relative to the mass of the resulting carrier. Therefore, a high encapsulation efficiency does not necessarily correspond to the highest loading capacity, particularly for hydrophilic compounds, whose localization in lipid-based systems is largely restricted to the aqueous phase and the interfacial regions of the vesicles [68,71,72,73,74,75].

The results of betanin encapsulation efficiency and loading capacity are presented in Table 3, while detailed explanations of the sample names are provided in Table 6. In the investigated formulations, EE% values depended on both the surfactant composition and the concentration of the incorporated betanin solution.

Samples containing the Tween 20/Mirasoft^®^ SL L60 mixture (xMixY) exhibited the highest encapsulation efficiency at the lowest concentration of betanin solution. For the H-C-yE-xMixY-1-xBe formulation containing 1.0% (*w*/*w*) betanin solution, EE% was 87.87 ± 3.22%. Increasing the concentration of betanin solution to 3.0% (*w*/*w*) reduced EE% to 36.70 ± 2.61%, whereas for the sample containing 5.0% (*w*/*w*) betanin solution, EE% increased to 62.53 ± 5.38%. These results indicate that the surfactant mixture promoted effective encapsulation of a small amount of betanin and further increases in the amount of active compound did not result in a linear increase in encapsulation efficiency. This may be attributed to partial saturation of the available aqueous and interfacial regions of the vesicles, as well as to structural reorganization of the system following an increase in betanin content [71,73].

A different relationship was observed for formulations containing Tween 20 as the sole surfactant (x2T). In this series, the most favorable encapsulation parameters were obtained at the intermediate concentration of betanin solution. The H-C-yE-x2T-1-yBe formulation containing 3.0% (*w*/*w*) betanin solution achieved an EE% of 85.37 ± 6.97%, whereas the corresponding values for samples containing 1.0% (*w*/*w*) and 5.0% (*w*/*w*) betanin solution were 49.00 ± 8.05% and 18.77 ± 3.43%, respectively. The pronounced decrease in EE% at the highest betanin concentration suggests that the encapsulation capacity of the system was exceeded. Under these conditions, part of the betanin may have remained outside the vesicular structures or may have been more weakly associated with the interfacial region, thereby reducing the efficiency of active-compound retention within the carrier [75,76,77].

Analysis of LC% provides additional information on the actual amount of betanin incorporated into the formulations (Table 3). The LC values ranged from 0.0029 ± 0.0002% to 0.0271 ± 0.0026% and indicated that an increase in the amount of betanin initially added did not always result in a proportional increase in loading capacity. In samples containing the Tween 20/Mirasoft^®^ SL L60 mixture, LC increased with increasing betanin solution concentration. The highest loading capacity was obtained for H-C-yE-xMixY-1-zBe sample, which contained 5.0% (*w*/*w*) betanin solution and exhibited an LC of 0.0271 ± 0.0026%. This value indicates that increasing the initial betanin concentration enabled retention of a greater absolute amount of the active compound within the carrier. At the same time, the EE% of this formulation was 62.53 ± 5.38%, meaning that it was not the sample with the highest encapsulation efficiency. This confirms that LC% and EE% were not maximized simultaneously. The relatively low loading capacity values obtained in the present study may be associated with the small initial amounts of betanin introduced into the formulations, as only 1.0%, 3.0%, or 5.0% (*w*/*w*) of an aqueous betanin solution [7.5 mg/mL] was incorporated during their preparation.

Among the formulations containing Tween 20 as the sole surfactant, the highest LC was obtained for H-C-yE-x2T-1-yBe sample, containing 3.0% (*w*/*w*) betanin solution, for which LC was 0.0199 ± 0.0014%. In contrast, the sample with the highest betanin concentration, H-C-yE-x2T-1-zBe, exhibited a lower LC of 0.0065 ± 0.0003%, despite the greater amount of active compound initially added. This relationship was consistent with the very low EE% of this formulation and indicates that, at 5.0% (*w*/*w*) betanin solution, the Tween 20-based system had reached or exceeded its encapsulation capacity. Consequently, further increasing the amount of betanin did not improve loading but instead reduced the efficiency of active-compound retention.

Such discrepancies between EE% and LC% are typical of vesicular systems containing hydrophilic compounds. Owing to its hydrophilic nature, betanin may be located primarily in the aqueous phase of the vesicles and, to some extent, within the interfacial region, where interactions with the polar moieties of phospholipids and surfactants may occur. A similar influence of carrier composition on betanin encapsulation efficiency has been demonstrated for nanoliposomes, in which chitosan surface modification affected particle size, PDI, zeta potential, encapsulation efficiency, betanin loading, stability, and bioavailability [46]. These findings confirm that the efficiency of betanin incorporation into lipid-based carriers depends not only on the amount of active compound added but also on the composition and organization of the vesicular structure.

A similar relationship was reported by Maestrelli et al. [78], who demonstrated that increasing the amount of active compound improved loading only until the maximum capacity of the ethosomes was reached. Beyond this threshold, further increases in active-compound concentration may reduce EE%, even when LC% remains relatively high. The results obtained in the present study are consistent with this mechanism and confirm that formulation optimization should consider EE% and LC% simultaneously, because improvement in one parameter does not necessarily result in improvement in the other. In addition, the loading capacity of ethosomes has been shown to be limited by lipid membrane composition, ethanol content, and the active-compound-to-lipid ratio. Once the optimal ratio of active compound to carrier components is exceeded, EE% may decrease despite an increase or maintenance of the absolute amount of encapsulated compound [79].

From the perspective of further formulation development, two systems were particularly favorable, although for different reasons. H-C-yE-xMixY-1-xBe sample exhibited the highest encapsulation efficiency, indicating highly effective retention of a small amount of betanin within the carrier. In contrast, H-C-yE-xMixY-1-zBe sample exhibited the highest loading capacity, corresponding to the greatest absolute amount of betanin within the system. The H-C-yE-x2T-1-yBe formulation represented a favorable compromise between high encapsulation efficiency and high loading. Therefore, the final selection of a formulation for further applications should consider not only EE% and LC%, but also colloidal stability, system homogeneity, and the betanin release profile.

It should be emphasized that the optimization criteria established during the initial screening were applied to betanin-free ethosomal formulations, whereas incorporation of betanin altered the physicochemical properties of the selected carrier systems. Consequently, the betanin-loaded formulations exhibited different combinations of encapsulation efficiency, loading capacity, particle-size homogeneity, and stability, and no single formulation simultaneously provided the most favorable values for all evaluated parameters. In particular, formulations exhibiting high encapsulation efficiency or loading capacity did not necessarily maintain the predefined PDI and hydrodynamic diameter criteria during refrigerated storage. Therefore, the investigated betanin-loaded systems should be regarded as formulations with distinct performance characteristics rather than as uniformly optimized carriers. Their final selection would require prioritization of the relevant quality attributes according to the intended application and further optimization of formulations exhibiting increased particle-size heterogeneity or changes in hydrodynamic diameter during storage.

### 2.10. SEM Morphology of Selected Formulations

Based on the results of physicochemical characterization, encapsulation efficiency, loading capacity, and release-profile analysis, selected formulations were subjected to additional morphological evaluation. SEM analysis was performed to qualitatively evaluate the particle morphology of the selected ethosomal systems. Both blank and betanin-loaded formulations were examined. Representative micrographs and particle-diameter distribution histograms obtained for samples analyzed 24 h (A) and 14 days (B) after preparation are presented in the Supplementary Materials (Figures S13–S20). Numerous clearly distinguishable spherical or nearly spherical structures were observed in all analyzed images. This morphology is consistent with the characteristics of lipid-based vesicular systems. At the same time, particles of different diameters were present in the individual samples, indicating the polydisperse nature of the investigated systems. The histograms were dominated primarily by fractions with diameters below 450 nm, whereas the proportion of structures larger than 600–750 nm was markedly lower. However, isolated larger objects with diameters of approximately 1000–1500 nm were observed in some formulations. These may have represented larger vesicles, particle clusters formed during drying, or local aggregates present in the analyzed samples.

Formulations containing the Tween 20/Mirasoft^®^ SL L60 mixture (MixY; Figures S13–S16) were characterized by numerous, predominantly regular and spherical structures. After 14 days of storage, their overall shape was preserved, indicating that the systems did not completely lose their morphological integrity during the investigated period. In the formulation containing 1% (*w*/*w*) betanin solution, a greater proportion of particles within the intermediate diameter ranges, mainly 151–450 nm, was observed after storage (Figure S13). In contrast, in systems containing 3% (*w*/*w*) and 5% (*w*/*w*) betanin solution, particles with diameters not exceeding approximately 450 nm predominated after 14 days, while the proportion of the largest objects was reduced compared with the images obtained after 24 h (Figures S14 and S15). These observations indicate that the surfactant mixture promoted preservation of the spherical particle morphology, although it did not completely eliminate particle-size heterogeneity.

Formulations containing Tween 20 as the sole surfactant (Figures S17–S20) also exhibited structures with a generally spherical morphology. However, their particle-size distribution was more dependent on betanin concentration and storage time. In the betanin-free sample and the formulation containing 1% (*w*/*w*) betanin solution, particles with diameters of up to approximately 450 nm predominated after 14 days, whereas the proportion of larger objects was relatively low. In particular, the formulation containing 1% (*w*/*w*) betanin solution exhibited a large number of small, regularly distributed structures, indicating a relatively homogeneous morphological appearance (Figure S17). More pronounced changes were observed for formulations containing 3% (*w*/*w*) and 5% (*w*/*w*) betanin solution. After 14 days of storage, an increased proportion of particles within the higher diameter ranges and a broader particle-size distribution were observed. In the sample containing 3% (*w*/*w*) betanin solution, structures exceeding 750 nm were present alongside numerous particles with diameters of 151–450 nm (Figure S18). The greatest heterogeneity was observed for the formulation containing 5% (*w*/*w*) betanin solution, in which both small particles and numerous larger objects within the 451–1000 nm range, as well as isolated structures of even greater dimensions, were present after 14 days (Figure S19). This morphology is consistent with the broad particle-size distribution of this formulation and may indicate an increased tendency of the particles to undergo reorganization or form local clusters during storage.

Comparison of the images obtained after 24 h and 14 days therefore indicates that the generally spherical particle morphology was preserved in all investigated formulations, whereas the degree of particle-size homogeneity depended on the surfactant type and the amount of incorporated betanin solution. Systems containing the Tween 20/Mirasoft^®^ SL L60 mixture generally exhibited a more stable morphological appearance, whereas selected formulations based exclusively on Tween 20, particularly those containing higher amounts of betanin, showed broader diameter distributions and a greater proportion of large structures.

The differences between the particle sizes observed by SEM and the D_h_ values determined by DLS result from the different sample-preparation and measurement conditions. DLS analyzes hydrated particles dispersed in suspension, whereas SEM requires sample drying, which may cause partial vesicle deformation, shrinkage, or aggregation.

### 2.11. In Vitro Release Profile and Release Kinetics of Betanin

**Figure 1:**
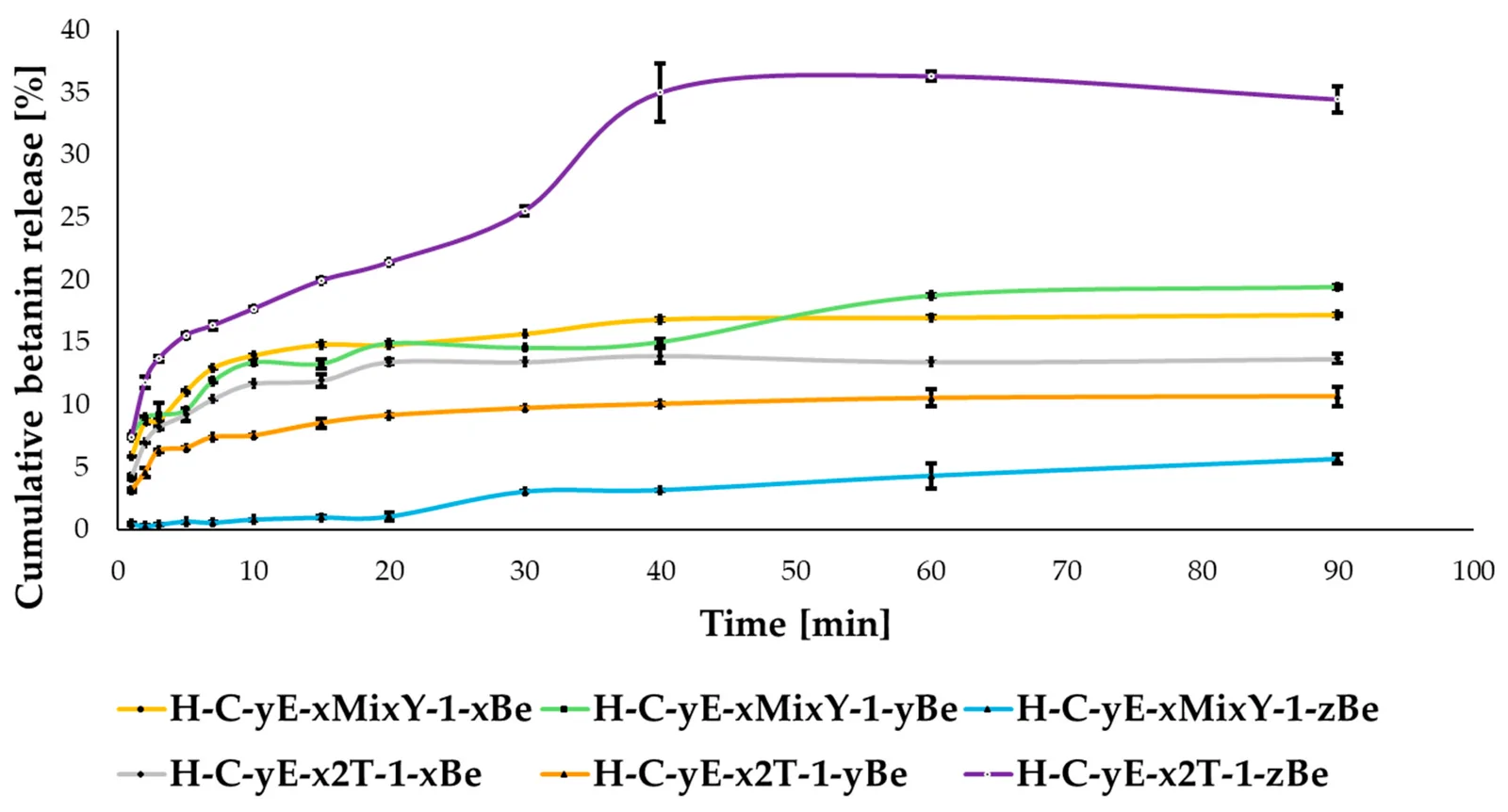
In vitro release profiles of betanin from ethosomal formulations in phosphate buffer at pH 5.8 over 90 min (±SD, *n* = 3). Formulations prepared with 10.0% (*w*/*w*) ethylene glycol, 30.0% (*w*/*w*) ethanol, 2.5% (*w*/*w*) phospholipid, 1.0% (*w*/*w*) cholesterol, and 0.625% (*w*/*w*) Tween 20/Mirasoft^®^ SL L60 surfactant mixture (xMixY) or Tween 20 (x2T). Samples were loaded with 1.0%, 3.0%, or 5.0% (*w*/*w*) of betanin solution [7.5 mg/mL] and prepared by 3 min homogenization (H). Detailed explanation of samples’ names in Table 6.

**Figure 2:**
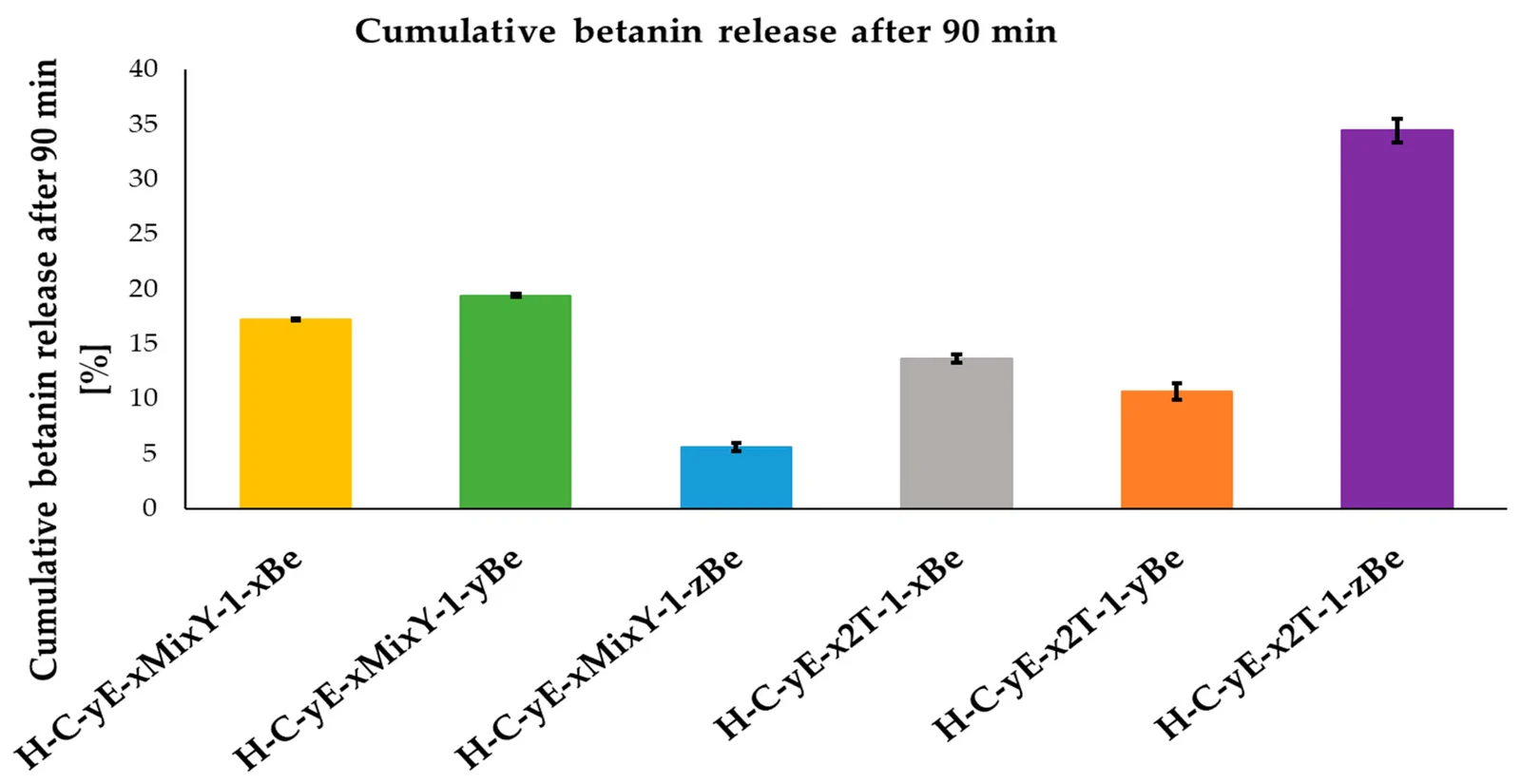
Cumulative betanin release from ethosomal formulations after 90 min in phosphate buffer with pH 5.8 (±SD, *n* = 3). Formulations prepared with 10.0% (*w*/*w*) ethylene glycol, 30.0% (*w*/*w*) ethanol, 2.5% (*w*/*w*) phospholipid, 1.0% (*w*/*w*) cholesterol, and 0.625% (*w*/*w*) Tween 20/Mirasoft^®^ SL L60 surfactant mixture (xMixY) or Tween 20 (x2T). Samples were loaded with 1.0%, 3.0%, or 5.0% (*w*/*w*) of betanin solution [7.5 mg/mL] and prepared by 3 min homogenization (H). Detailed explanation of samples’ names in Table 6.

Betanin release from the ethosomal formulations was evaluated in vitro in phosphate buffer at pH 5.8 over 90 min. The selection of a mildly acidic medium was justified by the intended dermal application of the investigated systems, as the skin surface is characterized by a slightly acidic pH [80]. After 90 min of testing, all formulations exhibited gradual and incomplete release profiles, with cumulative betanin release ranging from 5.67% to 34.49% (Figure 1 and Figure 2, Table 4). This indicates that a substantial proportion of the betanin remained retained within the ethosomal systems throughout the experiment. This finding confirms the ability of vesicular lipid carriers to retain a hydrophilic active compound and modulate its diffusion into the external medium [46,68,81]. For hydrophilic compounds, the release profile depends particularly on encapsulation efficiency, the distribution of the compound within the vesicular structure, and its ability to partition into the external phase [75].

The shapes of the release curves indicated that, for most samples, a rapid initial phase was followed by a marked decrease in the release rate (Figure 1). For samples H-C-yE-xMixY-1-xBe, H-C-yE-xMixY-1-yBe, H-C-yE-x2T-1-xBe, and H-C-yE-x2T-1-yBe, approximately 81%, 69%, 85%, and 71%, respectively, of the total amount released after 90 min was released within the first 10 min. This behavior may reflect the rapid transfer into the release medium of the betanin fraction located near the vesicle surface, present in the external aqueous phase, or more weakly associated with the interfacial region. After depletion of this more readily available fraction, the process slowed and may have been limited by diffusion from the vesicle interior and transport across the organized lipid-surfactant layer. A different release pattern was observed for H-C-yE-xMixY-1-zBe sample, for which the amount released after 10 min reached only 15% of the final value recorded after 90 min. In this sample, release increased slowly throughout the experiment, without a distinct initial burst. The H-C-yE-x2T-1-zBe formulation exhibited an intermediate profile: after 10 min, it reached approximately 51% of the final value, whereas a further pronounced increase occurred between 40 and 60 min. The slight decrease observed between 60 and 90 min should be interpreted as analytical variation around the plateau rather than as an actual reversal of the release process (Figure 1).

In the series of samples containing the mixture of Tween 20/Mirasoft^®^ SL L60, cumulative release after 90 min increased in the following order: H-C-yE-xMixY-1-zBe < H-C-yE-xMixY-1-xBe < H-C-yE-xMixY-1-yBe, reaching 5.67%, 17.23%, and 19.46%, respectively (Figure 2). This relationship was not proportional to the amount of betanin solution introduced. Increasing the proportion of betanin solution from 1.0 to 3.0% (*w*/*w*) resulted in only a slight increase in the final amount released, whereas a further increase to 5.0% (*w*/*w*) led to a more than threefold decrease in betanin availability in the release medium compared with the sample containing 3.0% (*w*/*w*) of active-compound solution. This indicates that, at the highest loading level, the relationship between the initial betanin content and its release from the xMixY system was not proportional. The strongest retention of betanin was observed for H-C-yE-xMixY-1-zBe. This formulation was also characterized by the highest loading capacity among all the investigated systems, with LC = 0.0271 ± 0.0026%, and an intermediate encapsulation efficiency, with EE = 62.53 ± 5.38% (Table 4). The low degree of release at a high LC value indicates that a greater absolute amount of betanin was effectively retained within the carrier structure but remained poorly accessible to the acceptor medium. Betanin may have been located in deeper regions of the ethosomes or may have interacted more strongly with the polar moieties of the phospholipids and surfactants. The presence of Mirasoft^®^ SL L60 may additionally have promoted the formation of a more compact and ordered interfacial region within the vesicles, thereby restricting the transport of the hydrophilic active compound into the external phase. In turn, the H-C-yE-xMixY-1-xBe sample exhibited the highest encapsulation efficiency among all six formulations, amounting to 87.87 ± 3.22%, with a low LC value of 0.0066 ± 0.0002% (Table 4). Nevertheless, only 17.23% of the betanin was released after 90 min. Thus, a high EE did not correspond to the highest extent of release, because this parameter describes the proportion of the retained compound relative to the amount initially added, but does not specify its absolute content or its location within the vesicular structure. At low loading, the available aqueous and interfacial domains may have retained a large proportion of the added betanin, while part of the compound may still have remained sufficiently close to the vesicle surface to diffuse into the acceptor medium during the initial phase of the release test. This case demonstrates that EE and LC should be interpreted jointly rather than as interchangeable measures of formulation quality [73].

A different behavior was observed in the series of ethosomal samples containing Tween 20 as the sole surfactant. The H-C-yE-x2T-1-xBe and H-C-yE-x2T-1-yBe formulations exhibited moderate release, reaching 13.73% and 10.71%, respectively, after 90 min (Figure 2). In the xBe sample containing 1% (*w*/*w*) betanin solution, EE was 49.00 ± 8.05% and LC was only 0.0029 ± 0.0002%. In contrast, in the yBe sample containing 3% (*w*/*w*) betanin solution, both parameters were markedly higher, reaching 85.37 ± 6.97% and 0.0199 ± 0.0014%, respectively (Table 4). The lower release observed for yBe sample, despite its high EE and LC, indicates that an active-compound loading provided more effective betanin retention in this system. It is possible that the use of 3% (*w*/*w*) betanin solution resulted in a favorable ratio between the amount of active compound and the available volume of the aqueous phase and interfacial area of the ethosomes. In contrast, the H-C-yE-x2T-1-zBe sample exhibited the highest cumulative betanin release among all the investigated systems, amounting to 34.49%. At the same time, this sample had the lowest EE value, equal to 18.77 ± 3.43%, and an LC of 0.0065 ± 0.0003%, despite the use of the highest amount of betanin solution during ethosome modification. This indicates that the retention capacity of the ethosomal system based exclusively on Tween 20 had been exceeded. A substantial proportion of the added betanin may have remained outside the stably formed vesicles, in the external aqueous phase, or in readily accessible interfacial regions, thereby facilitating its rapid transfer into the acceptor medium. Therefore, a high extent of release should not be interpreted as an unequivocally favorable carrier property, because it may coexist with low encapsulation efficiency and substantial dispersion heterogeneity.

Comparison of the two sample series prepared using Tween 20 alone or its mixture with the biosurfactant indicates that the effect of the amount of incorporated betanin on its release depended on the surfactant composition. At 5.0% (*w*/*w*) betanin solution, the Tween 20/Mirasoft^®^ SL L60 mixture limited release after 90 min to 5.67%, whereas the formulation containing Tween 20 alone released 34.49% of the betanin over the same period. The difference was therefore 28.82 percentage points. This finding indicates that the biosurfactant did not act solely as an additive stabilizing the colloidal properties, but also substantially altered the distribution and retention of betanin within the ethosomal vesicles. Similar effects of surface modification and interfacial-layer composition on the stability and release of betanin and other hydrophilic compounds have been reported for other lipid-based vesicular systems [82,83].

Because encapsulation efficiency, loading capacity, and active-compound release were determined for freshly prepared formulations, the interpretation of the relationships between carrier properties and release was based on the hydrodynamic diameter (D_h_), polydispersity index (PDI), and zeta potential (ZP) values measured on the day of sample preparation (Table 4). No simple relationship was observed in which any of the above-mentioned colloidal parameters independently determined the amount of betanin released after 90 min. Rather, the release profile resulted from the combined effects of surfactant composition, lipid-aqueous phase organization, active-substance loading, and the location of betanin within the system.

In the xMixY sample series prepared using the surfactant mixture, increasing the proportion of betanin solution from 1.0 to 3.0 and 5.0% (*w*/*w*) resulted in a progressive increase in hydrodynamic diameter (D_h_) after 24 h, from 153.07 ± 1.75 d.nm to 255.93 ± 7.58 d.nm and 279.03 ± 6.58 d.nm, respectively. At the same time, the PDI increased from 0.248 ± 0.001 to 0.349 ± 0.033 and 0.323 ± 0.009, respectively, whereas the corresponding ZP values were −26.77 ± 2.47 mV, −21.63 ± 0.21 mV, and −27.03 ± 0.25 mV. Despite these systematic changes in colloidal parameters, release after 90 min did not increase proportionally to the amount of betanin solution incorporated and amounted to 17.23%, 19.46%, and 5.67%, respectively (Table 4). Thus, the xMixY-zBe formulation exhibited the strongest betanin retention while also showing the highest loading capacity, with LC = 0.0271 ± 0.0026%. This indicates that, at the highest amount of incorporated betanin solution, the increase in hydrodynamic diameter and dispersion heterogeneity did not enhance the availability of the active compound but may instead have created conditions favoring its more effective retention in less accessible domains of the system. Moreover, within the xMixY series, the H-C-yE-xMixY-1-yBe formulation exhibited the highest betanin release after 90 min, reaching 19.46%, although its encapsulation efficiency was the lowest in this series, at 36.70 ± 2.61%. Its LC increased to 0.0094 ± 0.0007% but remained almost threefold lower than that of the corresponding zBe sample containing 5% (*w*/*w*) betanin solution. At 24 h after preparation, H-C-yE-xMixY-1-yBe sample was characterized by D_h_ = 255.93 ± 7.58 d.nm, PDI = 0.349 ± 0.033, and ZP = −21.63 ± 0.21 mV. This finding suggests that a relatively larger proportion of betanin may have remained in a more readily accessible aqueous phase, near the vesicle surface, or in a more weakly associated form. Comparison with the xMixY-xBe and xMixY-zBe samples confirms that neither the hydrodynamic diameter nor the homogeneity of the freshly prepared dispersion directly determined active-compound retention. It turned out that the location of betanin within the carrier and its interactions with the interfacial layer were also important [75,84,85]. Additionally, within the xMixY series, the xBe and yBe formulations released similar amounts of betanin despite marked differences in D_h_, PDI, and EE values. The H-C-yE-xMixY-1-xBe sample was the most homogeneous system, had lowest hydrodynamic diameter among all the investigated formulations, and exhibited the highest EE value of 87.87 ± 3.22%. However, the percentage of betanin released after 90 min was 17.23%. In contrast, H-C-yE-xMixY-1-yBe exhibited a higher hydrodynamic diameter, had a higher PDI value, and exhibited a markedly lower EE of 36.70 ± 2.61%, whereas its release after 90 min was only slightly higher, namely 19.46% (Table 4). This relationship confirms that the hydrodynamic diameter and homogeneity of the freshly prepared dispersion affect the characteristics of the system but do not directly determine the proportion of betanin located in a phase readily accessible to the acceptor medium.

In the series of samples containing Tween 20 as the sole surfactant (H-C-yE-x2T-1-), the hydrodynamic diameter after 24 h increased from 164.70 ± 5.15 d.nm for xBe to 225.30 ± 10.30 d.nm for yBe and 265.40 ± 9.16 d.nm for zBe (Table 4). The H-C-yE-x2T-1-zBe sample, with potentially highest loading of active-substance, showed the most distinct characteristics. One day after preparation, it exhibited the highest D_h_ value in the x2T series (265.40 ± 9.16 d.nm), the highest PDI among all the formulations (0.660 ± 0.013), and the most negative ZP value (−34.57 ± 0.40 mV). At the same time, it exhibited the lowest EE, at 18.77 ± 3.43%, and the highest release, at 34.49% (Table 4, Figure 2). This finding suggests that the increased heterogeneity of the vesicle population and the exceeded retention capacity of the Tween 20-based system favored the presence of a larger betanin fraction in the readily accessible aqueous phase or in more weakly associated interfacial regions. The highly negative zeta potential did not limit release, confirming that ZP primarily describes the electrostatic stabilization of the dispersion rather than membrane permeability or the location of the active compound.

For some of the formulations, the increase in hydrodynamic diameter observed during storage was accompanied by a noticeable decrease in PDI, indicating that the increase in hydrodynamic diameter was not associated with increased size heterogeneity. Instead, the vesicle population exhibited a narrower relative size distribution during storage. A similar phenomenon, characterized by an increase in D_h_ value accompanied by narrowing of the size distribution and a decrease in PDI, has been reported for lipid-based nanocarriers during storage [86]. In particular, Jakubek et al. described an increase in particle size accompanied by a decrease in PDI during storage of lipid nanoparticles, which was attributed to the evolution of the particle-size distribution during storage [87]. Comparable behavior has also been reported for ethosomes containing *Sambucus nigra* L. extract, for which an increase in D_h_ accompanied by a decrease in PDI was observed under selected storage conditions [88]. Therefore, the changes observed in the present study may reflect structural reorganization and/or partial fusion of vesicles and/or may be related to Ostwald-like ripening, resulting in larger vesicular structures with a narrower relative size distribution [89,90]. Such processes may alter the relative proportions of smaller and larger vesicles within the formulations, potentially contributing to the observed decrease in PDI. However, the available DLS results do not permit the explicit identification of the primary mechanism underlying these changes [91,92].

The most consistent interpretation was obtained when the formulation parameters were considered jointly in the context of EE and LC. High EE and LC values for H-C-yE-x2T-1-yBe were accompanied by limited release, whereas the low EE of H-C-yE-x2T-1-zBe was associated with the highest release after 90 min. In turn, H-C-yE-xMixY-1-zBe combined the highest LC with increased D_h_ values but released the smallest amount of betanin, indicating effective retention of a large absolute amount of the compound. Therefore, the results do not support the conclusion that smaller vesicles, a lower PDI, or a more negative ZP automatically increase or decrease release of active-substance. The organization of betanin within the system, which depended on surfactant type and carrier loading, was of key importance.

The observed relationships are consistent with reports on lipid-based betanin carriers demonstrating that vesicle composition and surface modification affect the stability, encapsulation efficiency, availability, and release profile of this compound [54,82]. In betanin-loaded nanoliposomes modified with chitosan, the surface layer affected particle size, PDI, zeta potential, stability, and the bioavailability of betanin, while also limiting its release from the carrier. These observations support the interpretation that the behavior of betanin in vesicular systems is strongly dependent on the organization and surface properties of the carrier [82].

The incomplete release of betanin observed over 90 min may be considered a potentially advantageous characteristic of the investigated ethosomal formulations, as effective retention of the active compound within the vesicular structures could limit premature release and contribute to prolonged delivery. However, excessive retention may also restrict the amount of betanin available for diffusion, highlighting the importance of balancing encapsulation efficiency with active-compound release. The interpretation of these findings is limited by the experimental conditions, particularly the relatively short release period and the use of an artificial diffusion membrane, which does not reproduce the complex barrier properties of human skin. Therefore, the present results demonstrate formulation-dependent encapsulation and in vitro release behavior but do not establish enhanced dermal penetration, skin retention, or transdermal bioavailability. Furthermore, although the physicochemical properties of the formulations were evaluated during 14 days of refrigerated storage, their long-term stability and biological safety, including cytotoxicity and skin compatibility, remain to be determined. Further investigations involving extended-release experiments, ex vivo skin permeation and retention studies, skin histological evaluation, and appropriate cytotoxicity assays are necessary to assess the suitability of the developed ethosomal systems for dermal applications. Moreover, the interpretation of the release profiles depends on the chemical stability of betanin under the experimental conditions and the maintenance of an adequate concentration gradient across the diffusion membrane. These factors should also be considered when interpreting the cumulative release values, particularly because membrane transport limitations or degradation of the released compound may affect the apparent release behavior.

**Figure 3:**
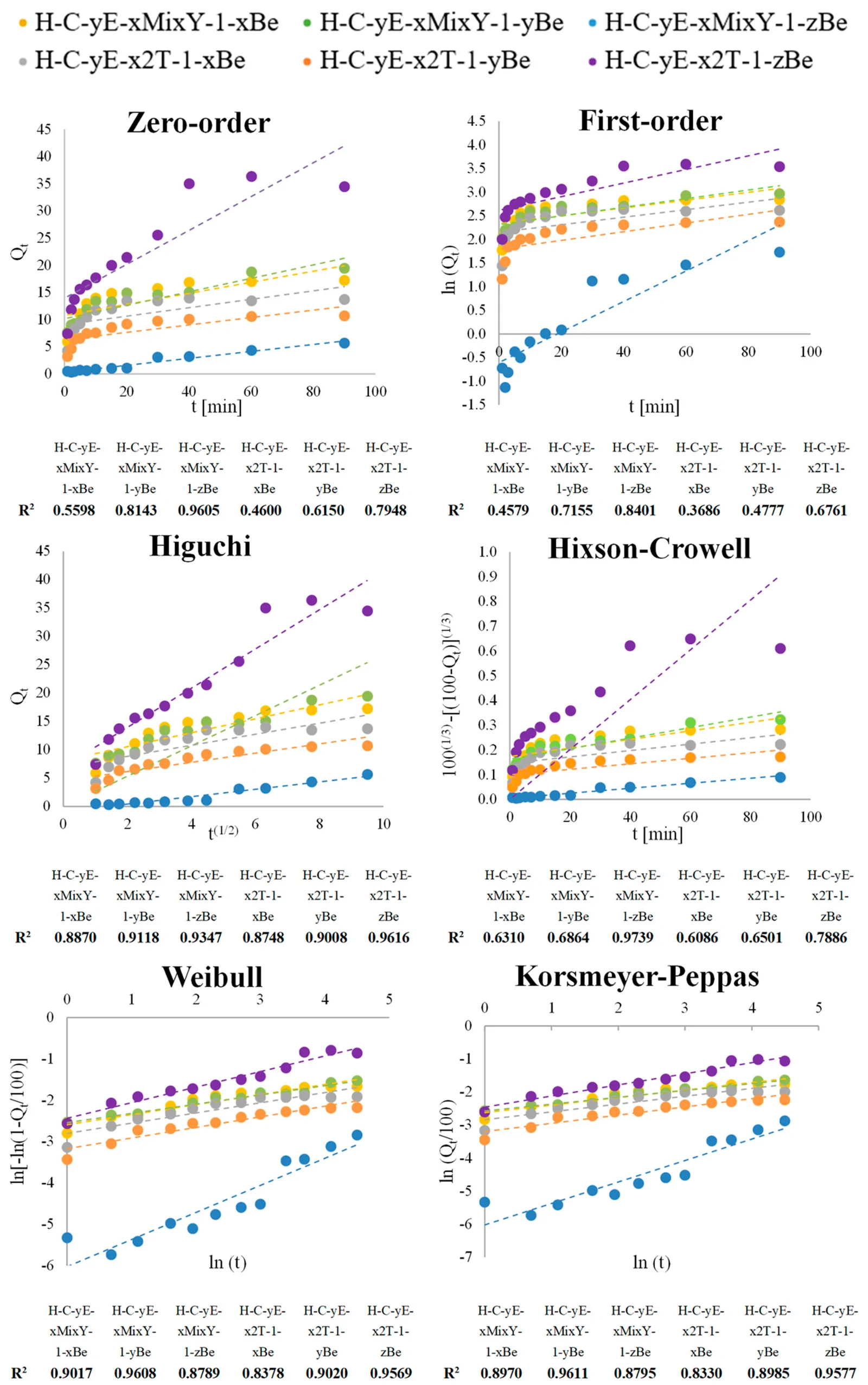
Experimental release profiles of betanin from the studied ethosomal formulations and their corresponding best-fit kinetic models. Symbols represent experimental data and dashed lines represent the best-fit model curves selected based on R^2^/AIC values, according to the model indicated in each panel. Formulations prepared with 10.0% (*w*/*w*) ethylene glycol, 30.0% (*w*/*w*) ethanol, 2.5% (*w*/*w*) phospholipid, 1.0% (*w*/*w*) cholesterol, and 0.625% (*w*/*w*) Tween 20/Mirasoft^®^ SL L60 surfactant mixture (xMixY) or Tween 20 (x2T). Samples were loaded with 1.0%, 3.0%, or 5.0% (*w*/*w*) of betanin solution [7.5 mg/mL] and prepared by 3 min homogenization (H). Detailed explanation of samples’ names in Table 6.

To characterize the release kinetics, the experimental data were linearized according to the equations of the zero-order, first-order, Higuchi, Hixson–Crowell, Korsmeyer–Peppas, and Weibull models. The application of multiple models enabled comparison of the release-profile shapes without presupposing a single dominant transport mechanism. The coefficients of determination (R^2^) were calculated in the spaces corresponding to the respective linear transformations, whereas the residual sum of squares (RSS), Akaike information criterion (AIC), and corrected Akaike information criterion (AICc) were calculated after back-transforming the predicted values to the original scale of cumulative release (Q_t_). The results obtained are presented in Figure 3 and Table 5.

Although the investigated ethosomal formulations are lipid-based vesicular systems rather than polymeric matrices, mathematical modeling of their release profiles provides a useful approach for comparing the time-dependent behavior of the encapsulated active compound. In the present study, the selected kinetic equations were applied primarily as empirical tools to characterize differences between the formulations and assess the suitability of mathematical functions for describing the experimental data. Their application was not intended to demonstrate polymer relaxation, swelling, erosion, or other mechanisms specific to polymer-based delivery systems. In particular, the Weibull model was used to compare the shapes of the release curves without assigning the observed behavior to a single physicochemical transport mechanism.

The Higuchi model was used to evaluate the relationship between the amount of released compound and the square root of time, whereas the Korsmeyer–Peppas model was applied as an empirical power-law equation enabling comparison of transport behavior based on the release exponent (*n*). Because cumulative betanin release did not exceed 60% for any of the analyzed formulations, all measurement points were included in the Korsmeyer–Peppas model fitting. The Weibull model was applied as a flexible empirical model capable of describing release profiles of different shapes without requiring assignment to a single, strictly defined physical mechanism [93,94]. For most formulations, the highest or comparable R^2^ values were obtained for the Weibull and Korsmeyer–Peppas models. The Weibull model was selected as a common empirical model to enable a standardized comparison across the entire series of samples. However, this does not imply that it was unequivocally the best statistical model for every formulation. In several cases, the differences in AICc values between the best-fitting equations were less than 2, indicating that these models may be regarded as statistically comparable.

For the H-C-yE-xMixY-1-xBe formulation, the Weibull model provided the highest R^2^ value, equal to 0.9017, and the lowest AICc value among the analyzed models. The Korsmeyer–Peppas model also described this profile well, with R^2^ = 0.8970. The difference in AICc between the two models was less than 2; therefore, the Weibull model may be considered preferable, although not unequivocally dominant, for describing betanin release kinetics from this formulation. For the H-C-yE-xMixY-1-yBe sample, the R^2^ values obtained for the Weibull and Korsmeyer–Peppas models were 0.9608 and 0.9611, respectively. The difference in AICc was very small, amounting to 0.059, with a slightly more favorable result for the Korsmeyer–Peppas model. These two models should therefore be considered practically equivalent. The use of the Weibull model in the overall comparison of the formulations is justified by its comparable fitting and its ability to provide a consistent framework for evaluating the entire series of samples. However, a different pattern was observed for betanin release from the H-C-yE-xMixY-1-zBe sample, which exhibited the lowest cumulative betanin release. The highest R^2^ value for this formulation was obtained with the zero-order model (R^2^ = 0.9605), which also showed the lowest AICc value (–20.46). Therefore, for this sample, the Weibull model should be regarded as a well-fitting common empirical model rather than as the unequivocally best statistical model.

In the series of samples containing Tween 20 as the sole surfactant, the Weibull model adequately described the release profiles of all three formulations. For H-C-yE-x2T-1-xBe and H-C-yE-x2T-1-yBe samples, the R^2^ values obtained with this model were 0.8378 and 0.9020, respectively. In both cases, the Weibull model yielded also the lowest AICc values. However, the differences relative to the Korsmeyer–Peppas model did not exceed 2 and this indicates that both equations provided comparable goodness of fit, with a slight advantage of the Weibull model according to the information criterion. For the H-C-yE-x2T-1-zBe sample, the Korsmeyer–Peppas model produced a slightly higher R^2^ value than the Weibull model, with values of 0.9577 and 0.9569, respectively. At the same time, the Weibull model was characterized by a lower AICc value, although the difference was only 0.595 (Table 5). Thus, these two models cannot be clearly distinguished on the basis of fitting. Nevertheless, application of the Weibull equation enables a consistent comparison of the release profiles across all formulations.

Furthermore, for all formulations, the Weibull shape parameter, β, was below 1 and ranged from 0.2223 to 0.6547. Such behavior corresponds to profiles in which the release rate is highest during the initial stage of the experiment and subsequently gradually decreases. However, the β parameter should not be directly equated with a specific physical mechanism because the Weibull model is empirical in nature [95,96,97,98]. The Korsmeyer–Peppas release exponent n_KP_ ranged from 0.2076 to 0.3354 for five formulations, whereas for H-C-yE-xMixY-1-zBe, the value of n_KP_ was as high as 0.6490. The higher value obtained for H-C-yE-xMixY-1-zBe indicates a different and more complex transport behavior, which may involve not only diffusion but also reorganization of the vesicular structure or changes occurring within the interfacial region. However, the interpretation of n_KP_ values should be approached with caution because the Korsmeyer–Peppas model was developed primarily for conventional polymeric systems, whereas the formulations analyzed in the present study were lipid-based vesicular systems [93,94,98].

**Figure 4:**
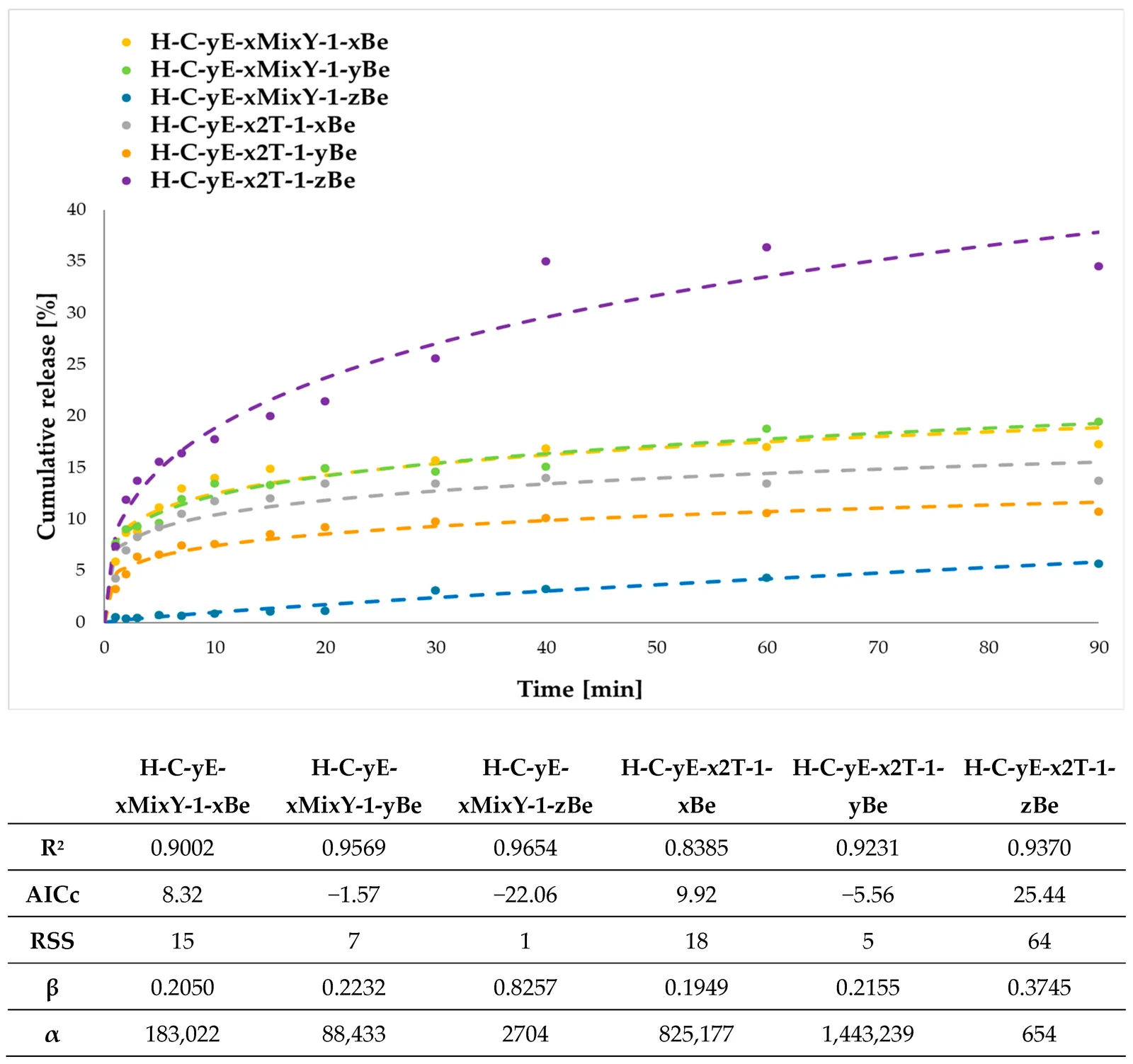
Experimental release profiles of betanin from the studied ethosomal formulations and their corresponding best-fit kinetic model (Weibull) with fitting parameters. Symbols represent experimental data and dashed lines represent the best-fit model curves selected based on R^2^/AIC. Formulations prepared with 10.0% (*w*/*w*) ethylene glycol, 30.0% (*w*/*w*) ethanol, 2.5% (*w*/*w*) phospholipid, 1.0% (*w*/*w*) cholesterol, and 0.625% (*w*/*w*) Tween 20/Mirasoft^®^ SL L60 surfactant mixture (xMixY) or Tween 20 (x2T). Samples were loaded with 1.0%, 3.0%, or 5.0% (*w*/*w*) of betanin solution [7.5 mg/mL] and prepared by 3 min homogenization (H). Detailed explanation of samples’ names in Table 6.

In the second stage of the release studies, the standard Weibull model was fitted directly to the untransformed data using nonlinear least squares approach. The asymptotic value was fixed at 100%, and the model parameters were constrained to positive values. The aim of this stage was to estimate the parameters of a common empirical model without distortions introduced by data transformation and to determine whether direct minimization of errors on the release scale improved the representation of the profiles. The nonlinear Weibull model fits to the mean experimental data points (*n* = 3) obtained in the release study are presented in Figure 4.

Direct fitting of the Weibull model adequately described all release profiles, yielding R^2^ values of 0.9002, 0.9569, 0.9654, 0.8385, 0.9231, and 0.9370 for H-C-yE-xMixY-1-xBe, H-C-yE-xMixY-1-yBe, H-C-yE-xMixY-1-zBe, H-C-yE-x2T-1-xBe, H-C-yE-x2T-1-yBe, and H-C-yE-x2T-1-zBe samples, respectively. The corresponding RSS values were 15, 7, 1, 18, 5, and 64%, whereas the AICc values were 8.32, −1.57, −22.06, 9.92, −5.56, and 25.44, respectively (Figure 4). These results confirm that the adequacy of the nonlinear fitting model was generally high, although it varied among the formulations. The lowest R^2^ was observed for H-C-yE-x2T-1-xBe, whereas the best fit was obtained for the slowly releasing H-C-yE-xMixY-1-zBe formulation.

Direct nonlinear fitting improved the representation of several profiles compared with the linearization-based procedure. For example, the AICc decreased from 11.17 to 8.32 for H-C-yE-xMixY-1-xBe, from 13.31 to 9.92 for H-C-yE-x2T-1-xBe, and from −1.58 to −5.56 for H-C-yE-x2T-1-yBe sample. For the H-C-yE-xMixY-1-yBe formulation, the values were almost identical (−1.56 and −1.57), as were those for H-C-yE-x2T-1-zBe (25.46 and 25.44) (Figure 4, Table 5). These differences confirm that logarithmic transformation may alter the error distribution, and therefore, the final interpretation of the α and β parameters was based on direct nonlinear fitting.

The Weibull shape parameter β obtained by nonlinear fitting ranged from 0.1949 to 0.8257 and remained below 1 for all formulations. For H-C-yE-xMixY-1-xBe, H-C-yE-xMixY-1-yBe, H-C-yE-x2T-1-xBe, and H-C-yE-x2T-1-yBe, the β values fell within the narrow range of 0.1949–0.2232. These values corresponded to strongly curved profiles in which the release rate was highest at the beginning and subsequently decreased gradually (Figure 4). For the H-C-yE-x2T-1-zBe sample, β was 0.3745, reflecting a more prolonged increase in release over time. The highest β value, (0.8257), was obtained for the H-C-yE-xMixY-1-zBe formulation, which is consistent with its markedly more gradual and approximately linear release profile over the investigated time range (90 min). The β parameter describes the shape of the curve but should not be directly attributed to a single transport mechanism [93,95,96,97,98]. Alternatively, the scale parameter α for the nonlinear Weibull model varied over a very wide range, from 653 min for the H-C-yE-x2T-1-zBe sample to approximately 1.44 × 10^6^ min for the H-C-yE-x2T-1-yBe sample. These high values resulted from fixing Q_∞_ at 100% despite incomplete release during the 90 min experiment. Thus, α parameter was largely extrapolated, extending beyond the observation period.

The difference in parameters arose because the linearized form of the model assigned different weights to individual data points, particularly at very low release values, whereas the nonlinear method minimized errors directly on the Q_t_ scale.

## 3. Materials and Methods

### 3.1. Chemicals

Phosphatidylcholine (Phospholipon^®^ 90G, Lipoid, Duisburg, Germany), ethanol (99.9%, P.P.H. Stanlab, Lublin, Poland), ethylene glycol (99%, Hadron Scientific, Kielce, Poland), cholesterol (Sigma-Aldrich, Darmstadt, Germany), and the following surfactants: Tween 20, Tween 80 (Sigma-Aldrich), and the Mirasoft^®^ SL L60 biosurfactant (Syensqo, Brussels, Belgium) were used to prepare the ethosomes. Betanin (99.9%, Liniaplus, Topolin, Poland) was used as the active compound and was prepared as an aqueous solution at a concentration of 7.5 mg/mL. Distilled water was used in all experiments.

The following reagents were used for antioxidant activity assays: DPPH (2,2-diphenyl-1-picrylhydrazyl, Sigma-Aldrich), ABTS (2,2′-azino-bis(3-ethylbenzothiazoline-6-sulfonic acid) diammonium salt (Sigma-Aldrich), potassium persulfate (Sigma-Aldrich), TPTZ (2,4,6-tris(2-pyridyl)-s-triazine; Thermo Fisher Scientific, Acros Organics, Waltham, MA USA), iron(III) chloride hexahydrate (Chempur, Piekary Śląskie, Poland), neocuproine (Sigma-Aldrich), anhydrous copper(II) chloride (Pol-Aura, Morąg, Poland), Folin–Ciocalteu reagent (Chempur), anhydrous sodium carbonate (Chempur), Trolox^®^ (Thermo Fisher Scientific, Acros Organics), anhydrous sodium acetate (Chempur), ammonium acetate (Chempur), and hydrochloric acid (35–38%, POCH Basic, Gliwice, Poland).

### 3.2. Preparation and Characterization of Beetroot Extract

The beetroot extract was prepared by ultrasound-assisted extraction according to the procedure described in a previous publication [99]. The extract was characterized using UV-Vis spectroscopy, LC-MS and GC-MS chromatography, and ATR-FTIR spectroscopy. In addition, qualitative and quantitative determination of betanin was performed using HPLC-UV-Vis. The HPLC-UV-Vis method showed excellent linearity (R^2^ = 1.0000), with a limit of detection (LOD) of 0.47 mg/mL and a limit of quantification (LOQ) of 1.41 mg/mL. In the present study, the extract was used exclusively to evaluate antioxidant properties and to justify the selection of betanin as a model active compound for encapsulation in ethosomes. The extract itself was not used in the preparation of the ethosomal formulations.

### 3.3. Determination of Antioxidant Activity

The antioxidant activity of the beetroot extract and an aqueous betanin solution (7.5 mg/mL) was evaluated using five complementary methods: DPPH, ABTS, CUPRAC, FRAP, and Folin–Ciocalteu assays. The use of methods based on different reaction mechanisms enabled a comprehensive assessment of the antioxidant properties of the samples.

Trolox^®^ calibration curves were prepared for all methods. Trolox^®^ is a commonly used reference substance that is a synthetic equivalent of vitamin E (tocopherol). It is used primarily to assess the antioxidant capacity of food products [100,101,102]. Previous antioxidant studies of betanin compounds using Trolox^®^ as a reference standard, have demonstrated its high antioxidant potential [103,104]. Trolox^®^ solutions at concentrations of 0.1, 0.2, 0.4, 0.6, 0.8, and 1.0 mmol/L were prepared by dissolving an accurately weighed amount of Trolox^®^ in several milliliters of ethanol and diluting to the required volume with distilled water. The results were expressed as Trolox^®^ equivalents (µmol TE/mL of sample). The assays were performed to confirm the high antioxidant activity of the beetroot extract and to support the selection of betanin as a model active compound for encapsulation in ethosomes.

#### 3.3.1. Determination of DPPH Radical-Scavenging Activity

Antioxidant activity was determined using the DPPH method according to procedures previously described in the literature [105,106,107]. The DPPH radical solution was prepared by dissolving 3.00 mg of DPPH in 100 mL of ethanol. The absorbance maximum was determined by UV-Vis spectrophotometry at a wavelength of 530 nm.

The extract and betanin samples were diluted with ethanol by adding 0.1 mL of each sample to 15 mL of ethanol and were subsequently mixed with the DPPH solution at a volume ratio of 1:1. Trolox^®^ standard solutions were incubated for 10 min, whereas the test samples were incubated for 60 min at room temperature under light-protected conditions. Absorbance was measured at 530 nm against the corresponding reference blanks. Radical-scavenging activity (RSA) was calculated according to Equation (1).

(1) $$\mathrm{Inhibition}\;\%=\left(\frac{A_{\mathrm{control}}-A_{\mathrm{sample}}}{A_{\mathrm{control}}}\right)\times 100\%$$

Formula used to calculate the percentage inhibition, where A_control_ is the absorbance of the control after background subtraction and A_sample_ is the absorbance of the sample after background subtraction.

#### 3.3.2. Determination of ABTS Radical-Scavenging Activity

Antioxidant activity was determined using the ABTS assay with the ABTS•^+^ radical cation. A 7.0 mM ABTS solution was prepared by dissolving 0.38380 g of ABTS diammonium salt in 100 mL of distilled water. Subsequently, a 2.24 mM potassium persulfate solution was prepared by dissolving 0.06623 g of K_2_S_2_O_8_ in 100 mL of distilled water. The ABTS•^+^ working solution was prepared by gently mixing 10 mL of the ABTS solution with 10 mL of the potassium persulfate solution. The mixture was incubated for 16 h at room temperature in the dark to allow the reaction to reach completion. Before analysis, the working solution was diluted with distilled water to obtain an absorbance of approximately 0.700 at 734 nm. A 10 µL aliquot of the sample and 3 mL of the diluted ABTS•^+^ solution were added to a polypropylene cuvette (12 × 12 × 45 mm). The mixture was incubated for 20 min at room temperature in the dark, after which the absorbance was measured at 734 nm. The diluted ABTS•^+^ solution without the sample added served as the control, whereas distilled water was used as the blank. Radical-scavenging activity (RSA) was calculated according to Equation (1).

#### 3.3.3. Determination of Reducing Capacity Using the CUPRAC Assay

The reducing capacity of the samples was determined using the CUPRAC assay according to the procedure described by Apak et al. [32]. A 0.01 M copper (II) chloride solution was prepared by dissolving 0.13445 g of CuCl_2_ in distilled water and adjusting the final volume to 100 mL. A 1.0 M sodium acetate buffer at pH 7.0 was prepared by dissolving 40.785 g of anhydrous sodium acetate in approximately 350 mL of distilled water and adding an acetic acid solution prepared from 0.213 g of acetic acid and approximately 50 mL of distilled water. The final volume was adjusted to 500 mL with distilled water.

A 0.0075 M neocuproine solution was prepared by dissolving 0.15620 g of neocuproine in ethanol and adjusting the final volume to 100 mL. The solution was protected from light.

A fresh reaction mixture was prepared immediately before analysis by combining 1.0 mL of 0.01 M CuCl_2_ solution, 1.0 mL of 0.0075 M neocuproine solution, 1.0 mL of sodium acetate buffer (1 M, pH 7.0), 0.5 mL of the sample, and 0.6 mL of distilled water. The mixture was incubated for 30 min at room temperature in the dark. Absorbance was subsequently measured at 450 nm using a polypropylene cuvette (12 × 12 × 45 mm). The control consisted of the reaction mixture in which the sample was replaced with distilled water, whereas sodium acetate buffer (1.0 M, pH 7.0) was used as the blank.

#### 3.3.4. Determination of Reducing Capacity Using the FRAP Assay

The FRAP assay was performed according to the method of Benzie and Strain [35], with modifications. First, a 0.3 M acetate buffer at pH 3.6 was prepared by mixing 0.28 M acetic acid and 0.02 M sodium acetate solutions at a volume ratio of 1:1. A 10 mM TPTZ solution in 40 mM HCl was then prepared by dissolving 0.031 g of TPTZ in 10 mL of HCl solution. A 20 mM iron (III) chloride solution was prepared by dissolving 0.541 g of FeCl_3_·6H_2_O in 100 mL of distilled water.

A fresh FRAP working solution was prepared by mixing 25 mL of acetate buffer (pH 3.6), 2.5 mL of TPTZ solution, and 2.5 mL of FeCl_3_·6H_2_O solution. The solution was heated to 37 °C in a water bath and protected from light. For the assay, 3.0 mL of the FRAP working solution was mixed with 100 µL of the sample in a polypropylene cuvette (12 × 12 × 45 mm) and incubated for 5 min at 37 °C. Absorbance was measured at 593 nm. The FRAP working solution without the sample served as the control, whereas 0.3 M acetate buffer at pH 3.6 was used as the blank.

#### 3.3.5. Determination of Total Reducing Capacity Using the Folin–Ciocalteu Assay

The total reducing capacity of the samples was determined using the Folin–Ciocalteu (F-C) assay. An aqueous Folin–Ciocalteu reagent solution was prepared by diluting the commercial reagent with distilled water at a ratio of 1:10. The solution was incubated for 60 min at room temperature in the dark in an amber glass bottle.

A 100 µL aliquot of the sample and 500 µL of the diluted Folin–Ciocalteu reagent were added to a measurement cuvette. After mixing, the mixture was incubated for 5 min at room temperature in the dark. Subsequently, 400 µL of a 7.5% (*w*/*w*) aqueous Na_2_CO_3_ solution was added. The mixture was mixed again and incubated for 30 min at room temperature. Absorbance was measured at 766 nm. Before analysis, the extract samples were diluted 1:10 with distilled water. The control consisted of a solution in which the sample was replaced with distilled water, whereas distilled water was used as the blank.

### 3.4. Research Design and Quality by Design (QbD)

**Figure 5:**
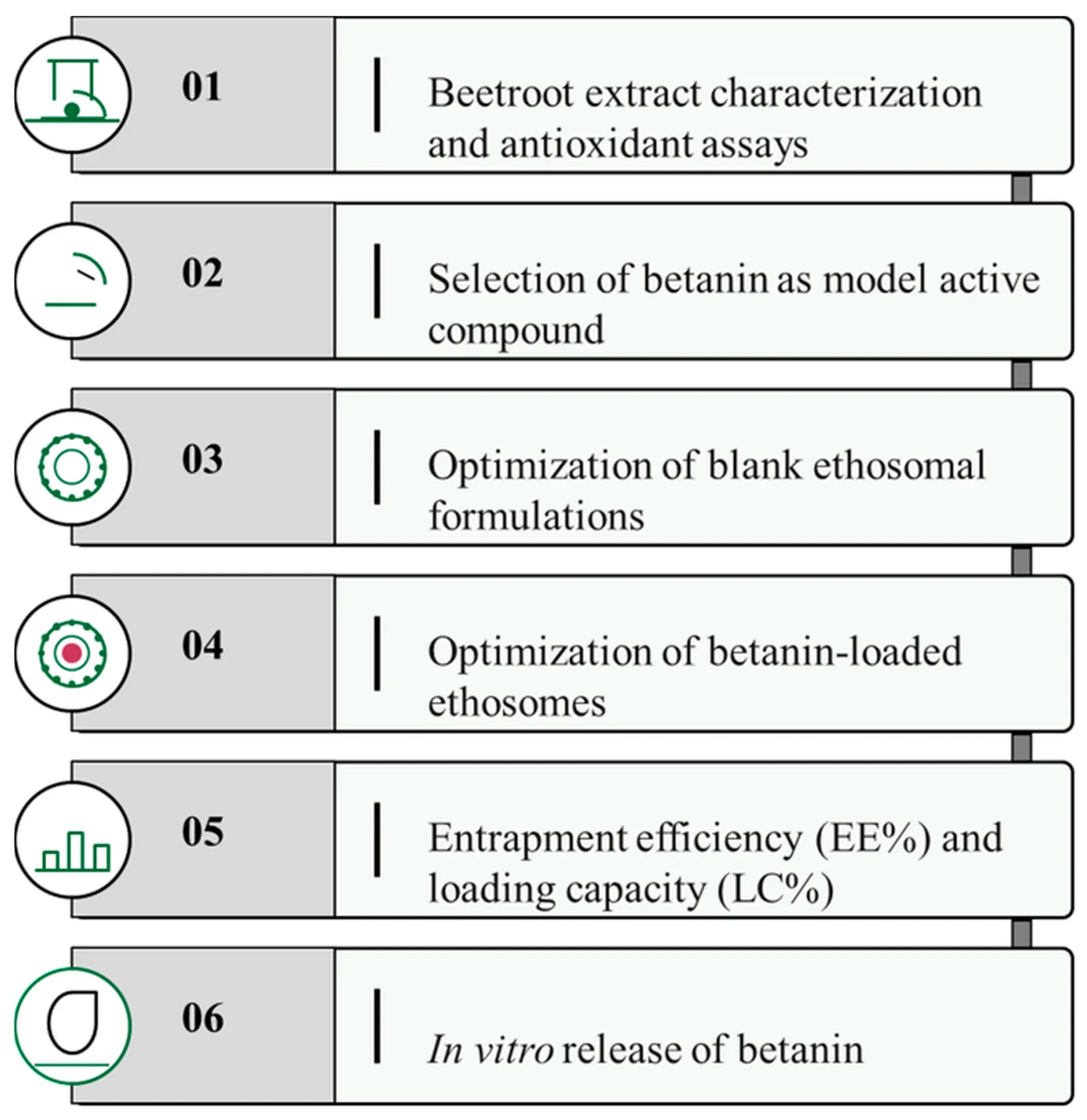
Schematic representation of the experimental workflow, including antioxidant evaluation, selection of betanin as a model active compound, optimization of blank and betanin-loaded ethosomal formulations, determination of entrapment efficiency and loading capacity, and in vitro release studies.

The study was guided by selected Quality by Design (QbD) principles, with emphasis on the identification of relevant formulation and process variables and on the predefined evaluation of critical quality attributes. A formal statistical Design of Experiments (DoE) was not applied; instead, formulation development was carried out using a sequential screening and optimization strategy. The main research hypothesis was that combining cholesterol with an appropriately selected surfactant or surfactant mixture containing the Mirasoft^®^ SL L60 biosurfactant would enable the development of ethosomal carrier systems with the desired physicochemical properties. A schematic overview of the successive stages of the study is presented in Figure 5.

In the first stage of the study, the antioxidant activity of beetroot extract and pure betanin was evaluated. These analyses were supplementary and were performed to support the selection of betanin as a model active compound for encapsulation. Subsequently, ethosomal formulations were developed and optimized by investigating the effects of the preparation method, surfactant type and concentration, biosurfactant incorporation, cholesterol content, and homogenization parameters on the properties of the resulting systems.

**Figure 6:**
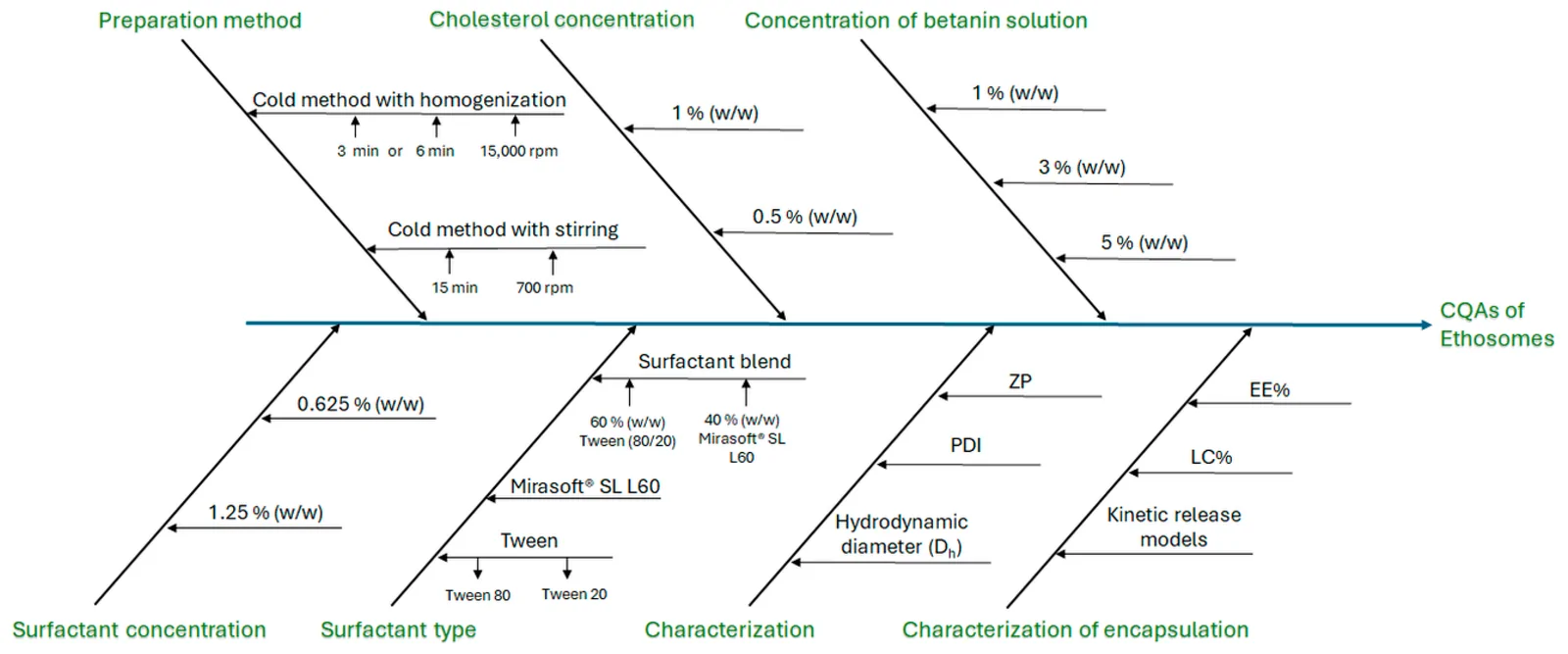
Ishikawa (fishbone) diagram used within the Quality by Design (QbD) approach to identify formulation and process variables affecting the critical quality attributes (CQAs) of the developed ethosomal formulations.

Hydrodynamic diameter (D_h_), polydispersity index (PDI), and zeta potential (ZP) were defined as the critical quality attributes (CQAs), as these parameters directly describe hydrodynamic size, dispersion homogeneity, and electrokinetic surface properties relevant to colloidal stability. ZP was considered as a complementary indicator of surface properties and colloidal stability, rather than as an independent measure of stability, because the formulations also contained nonionic surfactants capable of providing steric stabilization. Therefore, stability was interpreted jointly on the basis of ZP, D_h_, PDI, and their changes during storage. Factors potentially affecting the identified CQAs were presented using an Ishikawa diagram (Figure 6), which was employed as a qualitative tool to identify formulation and process variables potentially affecting the selected CQAs, rather than as part of a formal DoE analysis [108,109].

The scope of the study included the development, optimization, and physicochemical characterization of betanin-loaded ethosomes, as well as the evaluation of morphology, encapsulation efficiency, and the in vitro release profile. Skin penetration and retention were beyond the scope of the present study. Therefore, the results obtained relate to the formulation properties of the investigated systems and provide a basis for further biological studies.

### 3.5. Preparation and Optimization of Ethosomal Formulations

The formulations were optimized in a stepwise manner, with zeta potential (ZP), hydrodynamic diameter (D_h_), and polydispersity index (PDI) defined as the critical quality attributes (CQAs), as noted above. The effects of the preparation method, surfactant concentration, surfactant type, and cholesterol content on the physicochemical properties of the ethosomes were evaluated in successive stages. After identifying formulations that met the predefined quality criteria, betanin-loaded ethosomes were prepared.

Formulations were selected for further investigation if they met the following criteria: PDI ≤ 0.300, D_h_ < 300 nm, and ZP more negative than −25 mV. An additional selection criterion was the use of the lowest possible concentrations of excipients. These criteria were applied during the initial optimization of betanin-free ethosomal formulations to identify carrier compositions suitable for subsequent incorporation of the active compound. The selected thresholds were adopted as formulation-screening criteria, with particular emphasis on dispersion homogeneity, vesicle size, and electrostatic stability. Following betanin incorporation, the resulting formulations were characterized separately in terms of their physicochemical properties, encapsulation efficiency, loading capacity, and in vitro release behaviour. Consequently, the initial selection of the blank carriers should not be interpreted as confirmation that all betanin-loaded formulations maintained the predefined colloidal criteria.

All ethosomal formulations were prepared using the cold method. The organic-lipid phase was prepared by weighing the phospholipid, ethanol, ethylene glycol, surfactant, and, depending on the formulation, cholesterol into a tightly sealed vial. The mixture was then sonicated for 30 min (37 kHz; Fisherbrand FB11203, Fisher Scientific, Hampton, NH, USA). The aqueous phase consisted of distilled water or an aqueous betanin solution at the specified concentration. Both phases were heated to 30 °C, after which the aqueous phase was slowly added to the organic-lipid phase under stirring at 300 rpm (IKA, Staufen, Germany). The resulting ethosomal dispersions were either stirred at 700 rpm for 15 min or homogenized at 15,000 rpm for 3 or 6 min using an IKA Ultra-Turrax^®^ T25 easy clean homogenizer, according to the optimization plan.

The formulation coding system was based on the nomenclature presented in a previous publication [8]. The meaning of the individual symbols is summarized in Table 6.

**Table 6:** Nomenclature and composition codes used for ethosomal formulations.

Symbol	Meaning
1	30.0% (*w*/*w*) ethanol + 2.5% (*w*/*w*) phospholipid
C/xC	1.0% (*w*/*w*) or 0.5% (*w*/*w*) cholesterol
yE	10.0% (*w*/*w*) ethylene glycol
x8T/z8T	0.625% (*w*/*w*) or 1.25% (*w*/*w*) Tween 80
x2T/z2T	0.625% (*w*/*w*) or 1.25% (*w*/*w*) Tween 20
xMix	0.625% (*w*/*w*) surfactant mixture of Tween 80 and Mirasoft^®^ SL L60
xMixY	0.625% (*w*/*w*) surfactant mixture of Tween 20 and Mirasoft^®^ SL L60
H/yH	Homogenization: 3 min or 6 min; 15,000 rpm
xBe/yBe/zBe	1.0% (*w*/*w*) or 3.0% (*w*/*w*) or 5.0% (*w*/*w*) betanin solution [7.5 mg/mL]

The formulation codes indicate the homogenization conditions (H, 3 min; yH, 6 min), cholesterol content (C, 1.0%; xC, 0.5%), ethylene glycol incorporation (yE, 10.0%), surfactant composition (x2T, Tween 20; xMixY, Tween 20/Mirasoft^®^ SL L60), and the proportion of betanin solution added (xBe, 1.0%; yBe, 3.0%; zBe, 5.0%), while the symbol 1 denotes the base composition containing 30.0% ethanol and 2.5% phospholipid. Example: H-xC-yE-xMixY-1 denotes a sample prepared with the following composition: 2.5% (*w*/*w*) of phospholipid, 0.5% (*w*/*w*) of cholesterol, 10.0% (*w*/*w*) of ethylene glycol, 0.625% (*w*/*w*) of surfactant mixture of Tween 20 and Mirasoft^®^ SL L60, 30.0% (*w*/*w*) of ethanol; the preparation method used was homogenization: 3 min; 15,000 rpm.

#### 3.5.1. Effect of the Preparation Method on the Physicochemical Properties of Ethosomes

The effect of the preparation method on the physicochemical properties of cholesterol-containing ethosomes was evaluated using formulations containing 1.0% (*w*/*w*) cholesterol (C) and 1.25% (*w*/*w*) Tween 80 (z8T) or Tween 20 (z2T). Each formulation was prepared using three methods: the cold method with stirring, the cold method with homogenization for 3 min, and the cold method with homogenization for 6 min. The effect of the preparation method was assessed based on changes in hydrodynamic diameter (D_h_), polydispersity index (PDI), and zeta potential (ZP).

#### 3.5.2. Effect of Surfactant Concentration on the Physicochemical Properties of Ethosomes

After selecting the most favorable method for ethosome preparation, the effect of surfactant concentration on the colloidal properties of cholesterol-containing ethosomes was evaluated. Formulations containing 1.0% (*w*/*w*) cholesterol (C) and 0.625% (*w*/*w*) Tween 80 (x8T) or Tween 20 (x2T) were prepared. The formulations were obtained using the cold method with homogenization for 3 min (H) or 6 min (yH). The resulting systems were compared in terms of D_h_, PDI, and ZP to determine the effect of reducing the surfactant concentration on the stability and homogeneity of the ethosomal dispersions.

#### 3.5.3. Effect of Surfactant and Biosurfactant Type on the Physicochemical Properties of Ethosomes

In the subsequent stage of the study, the effect of surfactant type, including the use of a mixture of a synthetic surfactant and a biosurfactant, on the physicochemical properties of the ethosomes was evaluated. Formulations containing Tween 80 (xMix) or Tween 20 (xMixY), accounting for 60.0% (*w*/*w*) of the total surfactant content, and Mirasoft^®^ SL L60, accounting for 40.0% (*w*/*w*) of the total surfactant content, were prepared. The formulations were prepared in the presence of 1.0% (*w*/*w*) cholesterol (C) using the cold method with homogenization for 3 min (H) or 6 min (yH). Corresponding formulations without cholesterol were also prepared, allowing the effects of cholesterol and the surfactant mixtures on D_h_, PDI, and ZP to be evaluated.

#### 3.5.4. Effect of Cholesterol Concentration on the Physicochemical Properties of Ethosomes

The effect of reducing the cholesterol concentration on the properties of formulations containing surfactant mixtures was subsequently evaluated. For this purpose, formulations containing 0.5% (*w*/*w*) cholesterol (xC) and 0.625% (*w*/*w*) Tween 80/Mirasoft^®^ SL L60 (xMix) or Tween 20/Mirasoft^®^ SL L60 (xMixY) mixtures were prepared. The synthetic surfactant and biosurfactant accounted for 60.0% and 40.0% (*w*/*w*), respectively, of the total surfactant content. The formulations were prepared using the cold method with homogenization for 3 min (H) or 6 min (yH). The effect of cholesterol concentration was assessed based on changes in D_h_, PDI, and ZP.

#### 3.5.5. Preparation of Betanin-Loaded Ethosomes

Based on the optimization results, two formulations meeting the predefined quality criteria: PDI ≤ 0.300, D_h_ < 300 nm, and ZP < −25 mV were selected. An additional selection criterion was the use of the lowest possible concentrations of excipients.

Formulations containing 1.0% (*w*/*w*) cholesterol, 10.0% (*w*/*w*) ethylene glycol, 2.5% (*w*/*w*) phospholipid, 30.0% (*w*/*w*) ethanol, and either 0.625% (*w*/*w*) Tween 20 (x2T) or a Tween 20/Mirasoft^®^ SL L60 mixture (xMixY) were selected for further investigation. Both formulations were prepared using the cold method and homogenized for 3 min (H). An aqueous betanin solution at a concentration of 7.5 mg/mL was incorporated into each formulation at proportions of 1.0% (xBe), 3.0% (yBe), or 5.0% (zBe) (*w*/*w*). The percentages of 1.0%, 3.0%, and 5.0% (*w*/*w*) refer to the mass proportions of the aqueous betanin stock solution (7.5 mg/mL) incorporated into the final ethosomal formulations, rather than to the concentrations of purified betanin in the resulting dispersions.

### 3.6. Physicochemical Characterization and Stability Assessment of the Obtained Formulations

#### 3.6.1. Zeta Potential (ZP)

Zeta potential (ZP) was measured using a Zetasizer Nano-Z instrument (Malvern Panalytical, Malvern, UK) by electrophoretic light scattering (ELS) in phase analysis light scattering (PALS) mode. This technique enables the detection of small phase shifts in scattered light resulting from particle motion in an applied electric field.

Measurements were performed on the day of sample preparation, after 24 h, and after 14 days of refrigerated storage at 4 °C. Before analysis, 30 µL of each sample was diluted in 15 mL of distilled water. Measurements were conducted at 25 °C using a scattering angle of 173° [8]. Each measurement was performed in triplicate for the same sample, and the results are presented as the mean value ± SD.

#### 3.6.2. Determination of Hydrodynamic Diameter and Polydispersity Index

The hydrodynamic diameter (D_h_) and polydispersity index (PDI) were determined by dynamic light scattering (DLS) using a Zetasizer Nano-Z instrument (Malvern, UK). DLS enables the analysis of time-dependent fluctuations in the intensity of light scattered by a nanoparticle suspension as a result of Brownian motion.

A PDI value below 0.1 is generally considered indicative of a monodisperse system, whereas values ranging from 0.1 to 0.7 correspond to systems with a moderate degree of polydispersity. PDI values exceeding 0.7 are characteristic of highly polydisperse systems with a broad particle-size distribution. In the present study, PDI ≤ 0.300 was adopted as the threshold for acceptable dispersion homogeneity. The PDI threshold of 0.300 was adopted as an operational criterion for selecting comparatively homogeneous ethosomal dispersions during formulation optimization. Values exceeding this threshold were considered indicative of reduced particle-size homogeneity and were therefore regarded as unfavorable from the perspective of formulation optimization [110]. Nevertheless, such values do not necessarily indicate complete vesicle destabilization, and their interpretation should take into account the accompanying changes in hydrodynamic diameter and other physicochemical parameters.

The Zetasizer Nano-Z instrument employs non-invasive backscatter (NIBS) technology, in which the detector is positioned at an angle of 173°. This configuration provides high measurement sensitivity and enables the analysis of samples at relatively high concentrations. Owing to the design of the instrument, other scattering angles, including forward scattering, were not used. Measurements were performed using polystyrene cuvettes (10 × 10 × 45 mm; Sarstedt, Hildesheim, Germany).

Analyses were conducted on the day of sample preparation, after 24 h, and after 14 days of refrigerated storage at 4 °C. Before analysis, 30 µL of each sample was diluted in 15 mL of distilled water. The suitability of the resulting sample concentration for DLS analysis was verified using the instrument software, which confirmed that the measurement conditions were within the instrument-recommended range for reliable DLS analysis. No warnings indicating an unsuitable sample concentration were observed. The same dilution procedure was applied to all formulations and at all measurement time points to ensure comparability of the results. Nevertheless, because dilution with water decreases the ethanol concentration and modifies the composition of the continuous phase, a possible influence on vesicle organization and the absolute hydrodynamic diameter cannot be completely excluded. Therefore, the reported D_h_ values should primarily be interpreted comparatively under the standardized measurement conditions used in this study. Measurements were performed at 25 °C [8]. Each determination was carried out in triplicate for the same sample, and the results are presented as the mean value ± SD.

#### 3.6.3. Morphological Analysis of Ethosomal Formulations by Scanning Electron Microscopy (SEM)

The morphology of selected blank and betanin-loaded ethosomal formulations was evaluated using scanning electron microscopy (SEM). Sample images were obtained using an FEI Quanta 250 FEG scanning electron microscope (Thermo Fisher Scientific, Hillsboro, OR, USA). The samples were diluted with distilled water at a ratio of 40 µL of sample to 2 mL of water, deposited onto silicon substrates, dried at room temperature for 24 h, and imaged under high-vacuum conditions at an accelerating voltage of 5 kV.

Images were acquired for samples after 24 h and after 14 days of storage at 4 °C. Particle-size distributions based on the SEM images were analyzed using ImageJ 1.54g software (Java 1.8.0_345, 64-bit) and histograms were generated from the quantitative analysis of representative images for each sample.

### 3.7. Determination of Encapsulation Efficiency and Carrier Loading Capacity

Betanin encapsulation efficiency (EE%) and carrier loading capacity (LC%) were determined for betanin-loaded ethosomal formulations. Ethosome samples containing betanin solution (1 mL) were transferred into Eppendorf tubes and centrifuged for 30 min at 12,000 rpm (approximately 13,850× *g*) using a VWR Microstar 17 centrifuge (VWR, Radnor, PA, USA) equipped with a Thermo Scientific 75003424 rotor (Thermo Scientific, MA, Waltham, USA). After centrifugation, the supernatant was collected, and its absorbance was measured by UV-Vis spectrophotometry (Varian Cary 50 Conc spectrophotometer, Varian, Palo Alto, CA, USA) at λ_max_ = 538 nm, corresponding to the absorption maximum of betanin. The same centrifugation conditions were applied to all formulations. However, complete sedimentation of nanoscale ethosomal vesicles was not independently verified using an orthogonal separation method. Therefore, the possibility that a fraction of the smallest vesicles remained in the supernatant cannot be completely excluded, which may lead to an underestimation of the absolute EE% values. Consequently, EE% and LC% should primarily be interpreted comparatively among formulations analyzed using the same separation protocol.

The concentration of free, non-encapsulated betanin was determined using a calibration curve prepared for betanin in water. The UV-Vis calibration curve exhibited linear behavior over the concentration range used for the determination. However, a full formal validation of this spectrophotometric procedure, including independent assessment of accuracy, precision, specificity, limits of detection and quantification, and analyte stability, was not performed as a separate study. Encapsulation efficiency (EE%) and carrier loading capacity (LC%) were calculated according to Equations (2) and (3) [111,112,113]:

(2) $$\mathrm{EE}\%=\left[\frac{\left(C_{\mathrm{total}}-C_{\mathrm{free}}\right)}{C_{\mathrm{total}}}\right]\times 100\%$$

(3) $$\mathrm{LC}\%=\left[\frac{\left(C_{\mathrm{total}}\times \mathrm{EE}\%\right)}{\mathrm{carrier}\;\mathrm{mass}}\right]\times 100\%$$

where EE% is the encapsulation efficiency; C_total_ is the concentration of betanin initially introduced [mg/mL]; C_free_ is the concentration of free betanin in the solution [mg/mL]; and LC% is the loading capacity.

Equations used to calculate the encapsulation efficiency (EE%, (2)) and carrier loading capacity (LC%, (3)) of betanin-loaded ethosomes.

### 3.8. In Vitro Study of Betanin Release

Betanin release from selected ethosomal formulations was evaluated in vitro using a diffusion system with a Cuprophan membrane as the separating barrier. An aliquot of the ethosomal sample (0.4 mL) prepared using an aqueous betanin stock solution at a concentration of 7.5 mg/mL was introduced into a dedicated diffusion cell, the contact surface of which was covered with a Cuprophan membrane (Agilent Technologies, Santa Clara, CA, USA). The cell was then immersed in 85 mL of phosphate buffer at pH 5.8, which served as the release medium. The volume of the acceptor medium (85 mL) was selected to provide a substantial dilution of the released betanin and maintain a concentration gradient across the diffusion membrane. Phosphate buffer at pH 5.8 was selected to provide a mildly acidic release environment relevant to the intended dermal application of the formulations. This pH is also within the range reported in the literature to favor the physicochemical stability of betanin [114]. The 90 min experimental period was used to compare the initial release behavior of the investigated formulations under identical conditions. Accordingly, the release experiments were intended to characterize short-term, formulation-dependent differences in betanin transfer into the acceptor medium rather than to establish complete or prolonged release.

The experiment was conducted at room temperature under magnetic stirring at 500 rpm for 90 min. At predetermined time points, samples of the release medium were collected, and the betanin concentration was determined by UV-Vis spectrophotometry at λ_max_ = 538 nm. After each measurement, the sampled medium was returned to the system. The amount of released betanin was calculated using a calibration curve prepared for betanin in phosphate buffer at pH 5.8. The calibration curve exhibited linear behavior over the concentration range used for the release measurements.

The release data were fitted to selected kinetic models using linear regression. Linearized forms of the following models were applied: zero-order, first-order, Higuchi, Korsmeyer–Peppas, Hixson–Crowell, and Weibull [94]. In addition, the experimental data were fitted directly to the Weibull model using nonlinear regression. The following parameters: R^2^, k, n, α, β as well as RSS, AIC, AICc were determined and used to assess the goodness of fit of each model to the experimental data [98,115,116,117]. The equations used for the calculations are provided in Table S10, along with the corresponding references [118,119,120,121,122,123,124,125,126].

### 3.9. Statistical Analysis

Statistical analysis was performed to evaluate the effects of storage time and formulation composition on the physicochemical properties of the investigated samples. The analyzed parameters included zeta potential (ZP), hydrodynamic diameter (D_h_), and polydispersity index (PDI). Measurements were performed for freshly prepared samples and after 24 h and 14 days of storage at 4 °C. Each experimental group comprised three independent measurements.

Before selecting the statistical test, the distribution of the data within each group was assessed using the Shapiro–Wilk test, whereas homogeneity of variances was evaluated using Levene’s test. When the assumptions of normality and homogeneity of variances were met, differences between groups were analyzed using one-way analysis of variance (one-way ANOVA). When the assumptions required for one-way ANOVA were not satisfied, the nonparametric Kruskal–Wallis test was applied.

Two types of comparisons were performed: comparison of the same sample over time and comparison of different samples at the same time point. When the omnibus test indicated statistically significant differences, appropriate post hoc analyses were conducted. Tukey’s honestly significant difference (HSD) post hoc test was used for parametric data, whereas multiple comparisons of mean ranks with Bonferroni correction were performed for nonparametric data. Differences were considered statistically significant at *p* < 0.05.

Because of the limited number of replicates in each group, the results of the normality tests were interpreted with caution. Therefore, the final interpretation of the statistical results was additionally supported by analysis of the mean values, standard deviations, and the overall direction of the observed changes.

## 4. Conclusions

The results demonstrated that the physicochemical properties of the investigated ethosomal formulations were strongly influenced by surfactant composition, cholesterol content, and homogenization conditions. The initial optimization enabled the selection of two betanin-free ethosomal compositions with favorable colloidal properties. Formulations containing 2.5% (*w*/*w*) phospholipid, 30.0% (*w*/*w*) ethanol, 10.0% (*w*/*w*) ethylene glycol, 1.0% (*w*/*w*) cholesterol, and 0.625% (*w*/*w*) Tween 20 or Tween 20/Mirasoft^®^ SL L60, prepared using 3 min homogenization, exhibited the most favorable colloidal properties under the investigated conditions. However, following incorporation of betanin, the formulations exhibited different combinations of encapsulation efficiency, loading capacity, particle-size homogeneity, and release behavior, indicating that no single formulation simultaneously satisfied all the investigated performance criteria. The highest encapsulation efficiency reached 87.87 ± 3.22%, whereas the maximum loading capacity was 0.0271 ± 0.0026%. In vitro release was incomplete and formulation-dependent, ranging from 5.67% to 34.49% after 90 min at pH 5.8. These results indicate that the composition of the ethosomal systems substantially affected betanin retention and release behavior.

The present study establishes the physicochemical characteristics, short-term storage stability, encapsulation performance, and in vitro release profiles of the developed betanin-loaded ethosomes. However, their dermal delivery potential remains unconfirmed because skin permeation, skin retention, and biological safety were not evaluated. Further investigations are required to determine the long-term stability, skin compatibility, and biological performance of these formulations.

## Figures and Tables

**Table 1:** Comparison of lipid-based vesicular carriers.

Carrier	Composition	Characteristic	Ref.
Liposomes	Lipid	Vesicles composed of a phospholipid bilayer	[6,7,8]
Ethosomes	Lipid + ethanol	Modified lipid vesicles containing a significant amount of ethanol	[7,9,10]
Transethosomes	Lipid + ethanol + edge activator (for example surfactant)	Ethosomes that also contain an ingredient that increases elasticity	[10,11,12]
Phytosomes	Lipid + phytochemicals	Complexes of plant substances/phytochemicals with phospholipids	[13,14,15]

**Table 2:** Antioxidant activity of beetroot extract and betanin solution expressed as Trolox^®^ equivalents (µmol TE/mL of sample).

Sample	DPPH		ABTS		CUPRAC		FRAP		Folin–Ciocalteu	
	µmol TE/mL	±SD	µmol TE/mL	±SD	µmol TE/mL	±SD	µmol TE/mL	±SD	µmol TE/mL	±SD
Beetroot extract	165.9	0.0	148.7	0.2	14.1	0.1	28.8	0.0	14.9	0.1
Betanin [7.5 mg/mL]	130.1	0.7	226.5	0.0	3.2	0.0	12.9	0.0	89.4	0.2

**Table 3:** Encapsulation efficiency (EE%), loading capacity (LC%), and calculated amount of betanin solution [7.5 mg/mL] or pure active compound in 1 g of betanin-loaded ethosomal formulations. Formulations prepared with 10% (*w/w*) ethylene glycol, 30% (*w*/*w*) ethanol, 2.5% (*w*/*w*) phospholipid, 1% (*w*/*w*) cholesterol, and 0.625% (*w*/*w*) Tween 20/Mirasoft^®^ SL L60 surfactant mixture (xMixY) or Tween 20 (x2T). Samples loaded with 1.0, 3.0 or 5.0% (*w*/*w*) betanin solution [7.5 mg/mL] and prepared by 3 min homogenization (H). Results presented as mean value ± SD. Sample names explained in Table 6.

Formulation	Betanin Solution [7.5 mg/mL] Added to the Formulation [%] (*w*/*w*)	Amount of Betanin Solution [7.5 mg/mL] in 1 g of Ethosomes [mg]	Amount of Pure Betanin in 1 g of Ethosomes [mg] *	EE% ± SD [%]	LC% ± SD [%]
H-C-yE-xMixY-1-xBe	1	10	0.075	87.87 ± 3.22	0.0066 ± 0.0002
H-C-yE-xMixY-1-yBe	3	30	0.225	36.70 ± 2.61	0.0094 ± 0.0007
H-C-yE-xMixY-1-zBe	5	50	0.375	62.53 ± 5.38	0.0271 ± 0.0026
H-C-yE-x2T-1-xBe	1	10	0.075	49.00 ± 8.05	0.0029 ± 0.0002
H-C-yE-x2T-1-yBe	3	30	0.225	85.37 ± 6.97	0.0199 ± 0.0014
H-C-yE-x2T-1-zBe	5	50	0.375	18.77 ± 3.43	0.0065 ± 0.0003

* The amount of pure betanin was calculated from the volume of betanin solution added [7.5 mg/mL], taking into account the solution density of 988.7 [mg/mL].

**Table 4:** Summary of Physicochemical Parameters for Samples Modified with Betanin Solution.

Formulation	D_h_ [d.nm]		PDI		ZP [mV]		EE%	LC%	Release After 90 min [%]
	After 24 h	After 14 Days	After 24 h	After 14 Days	After 24 h	After 14 Days			
H-C-yE-xMixY-1-xBe	153.07 ± 1.75	239.70 ± 10.34	0.248 ± 0.001	0.315 ± 0.004	−26.77 ± 2.47	−22.23 ± 0.64	87.87 ± 3.22	0.0066 ± 0.0002	17.23
H-C-yE-xMixY-1-yBe	255.93 ± 7.58	325.77 ± 2.81	0.349 ± 0.033	0.145 ± 0.003	−21.63 ± 0.21	−24.13 ± 0.15	36.70 ± 2.61	0.0094 ± 0.0007	19.46
H-C-yE-xMixY-1-zBe	279.03 ± 6.58	416.63 ± 10.39	0.323 ± 0.009	0.269 ± 0.010	−27.03 ± 0.25	−30.10 ± 3.05	62.53 ± 5.38	0.0271 ± 0.0026	5.67
H-C-yE-x2T-1-xBe	164.70 ± 5.15	340.10 ± 15.70	0.181 ± 0.018	0.380 ± 0.013	−28.00 ± 0.53	−29.93 ± 0.21	49.00 ± 8.05	0.0029 ± 0.0002	13.73
H-C-yE-x2T-1-yBe	225.30 ± 10.30	339.50 ± 2.55	0.282 ± 0.015	0.261 ± 0.013	−28.20 ± 0.82	−27.90 ± 0.36	85.37 ± 6.97	0.0199 ± 0.0014	10.71
H-C-yE-x2T-1-zBe	265.40 ± 9.16	248.50 ± 6.51	0.660 ± 0.013	0.519 ± 0.027	−29.90 ± 0.17	−34.57 ± 0.40	18.77 ± 3.43	0.0065 ± 0.0003	34.49

**Table 5:** Summary of RSS, AIC, AICc, and R^2^ parameters, as well as the k, n, α, and β coefficients, for all investigated samples and kinetic models in linear fitting.

H-C-yE-xMixY-1-xBe								
Model	RSS	AIC	AICc	R^2^	Parametr 1		Parametr 2	
Zero-order	68	24.80	26.13	0.5598	k_0_	0.1018		
First-order	88	27.97	29.31	0.4579	k_1_	0.0083		
Higuchi	252	38.54	38.94	0.7676	k_H_	2.6447		
Hixson–Crowell	797	52.35	52.75	0.5686	k_HC_	0.0050		
Korsmeyer–Peppas	21	10.88	12.21	0.8970	k_KP_	0.0724	n_KP_	0.2276
Weibull	20	9.83	11.17	0.9017	β	0.2422	α	44,184
**H-C-yE-xMixY-1-yBe**								
**Model**	**RSS**	**AIC**	**AICc**	**R^2^**	**Parametr 1**		**Parametr 2**	
Zero-order	29	14.63	15.96	0.8143	k_0_	0.0012		
First-order	44	19.61	20.94	0.7155	k_1_	0.0092		
Higuchi	195	35.47	35.87	0.9299	k_H_	2.6700		
Hixson–Crowell	679	50.43	50.83	0.8233	k_HC_	0.0052		
Korsmeyer–Peppas	7	−2.95	−1.62	0.9611	k_KP_	0.0762	n_KP_	0.2076
Weibull	7	−2.89	−1.56	0.9608	β	0.2223	α	92,206
**H-C-yE-xMixY-1-zBe**								
**Model**	**RSS**	**AIC**	**AICc**	**R^2^**	**Parametr 1**		**Parametr 2**	
Zero-order	1	−21.79	−20.46	0.9605	k_0_	0.0639		
First-order	23	11.96	13.29	0.3421	k_1_	0.0323		
Higuchi	5	−8.75	−8.35	0.8616	k_H_	0.4976		
Hixson–Crowell	2	−19.83	−19.43	0.9451	k_HC_	0.0011		
Korsmeyer–Peppas	4	−9.78	−8.45	0.8925	k_KP_	0.0024	n_KP_	0.6490
Weibull	4	−9.75	−8.42	0.8923	β	0.6547	α	9878
**H-C-yE-x2T-1-xBe**								
**Model**	**RSS**	**AIC**	**AICc**	**R^2^**	**Parametr 1**		**Parametr 2**	
Zero-order	59	23.07	24.40	0.4600	k_0_	0.0775		
First-order	74	25.81	27.14	0.3686	k_1_	0.0080		
Higuchi	193	35.32	35.72	0.8748	k_H_	2.1814		
Hixson–Crowell	585	48.65	49.05	0.6086	k_HC_	0.0040		
Korsmeyer–Peppas	25	12.75	14.08	0.8330	k_KP_	0.0588	n_KP_	0.2349
Weibull	23	11.98	13.31	0.8378	β	0.2466	α	86,996
**H-C-yE-x2T-1-yBe**								
**Model**	**RSS**	**AIC**	**AICc**	**R^2^**	**Parametr 1**		**Parametr 2**	
Zero-order	24	12.43	13.76	0.6150	k_0_	0.0681		
First-order	34	16.45	17.78	0.4777	k_1_	0.0092		
Higuchi	80	24.80	25.20	0.9008	k_H_	1.6041		
Hixson–Crowell	278	39.69	40.09	0.6501	k_HC_	0.0030		
Korsmeyer–Peppas	7	−2.18	−0.85	0.8985	k_KP_	0.0410	n_KP_	0.2476
Weibull	7	−2.91	−1.58	0.9020	β	0.2566	α	236,052
**H-C-yE-x2T-1-zBe**								
**Model**	**RSS**	**AIC**	**AICc**	**R^2^**	**Parametr 1**		**Parametr 2**	
Zero-order	209	38.29	39.62	0.7948	k_0_	0.3116		
First-order	459	47.73	49.06	0.6761	k_1_	0.0143		
Higuchi	248	38.35	38.75	0.9168	k_H_	4.6840		
Hixson–Crowell	1272	57.96	58.36	0.8039	k_HC_	0.0101		
Korsmeyer–Peppas	67	24.72	26.05	0.9577	k_KP_	0.0856	n_KP_	0.3345
Weibull	64	24.12	25.46	0.9569	β	0.3759	α	653

## Data Availability

The raw data supporting the conclusions of this article are available from the corresponding authors upon reasonable request.

## Supplementary Materials

The following supporting information can be downloaded at https://www.mdpi.com/article/10.3390/ijms27219548/s1.
