# Supplementary material for: Cholesterol- and Biosurfactant-Stabilized Ethosomes for Betanin: Formulation, Encapsulation and Controlled In Vitro Release
Source: Int J Mol Sci. 2026 Oct 26;27(21):9548. doi: 10.3390/ijms27219548 (PMC13762820; doi:10.3390/ijms27219548)
Supplement: Supplementary file 1 [file ijms-27-09548-s001.zip › ijms-4604944-supplementary.pdf]

SUPPLEMENTARY MATERIALS

Cholesterol- and biosurfactant-stabilized ethosomes for betanin: formulation, encapsulation and controlled in vitro release

Jagoda Chudzińska-Skorupinska, Agata Wawrzyńczak* and Agnieszka Feliczak-Guzik*

Adam Mickiewicz University in Poznań, Faculty of Chemistry, Department of Applied Chemistry; Uniwersytetu Poznańskiego 8, 61-614 Poznań, Poland; jagoda.chudzinska@amu.edu.pl, agata.wawrzynczak@amu.edu.pl, agnieszka.feliczak-guzik@amu.edu.pl.

* Correspondence: agaguzik@amu.edu.pl; agata.wawrzynczak@amu.edu.pl

Table S1. Percentage inhibition of samples in the DPPH and ABTS antioxidant assays.

	DPPH [%]	ABTS [%]
Beetroot extract	59.39	20.64
Betanin [7.5 mg/mL]	53.89	35.04

Table S2. Zeta potential values of ethosomal formulations containing 10.0 % (w/w) ethylene glycol (yE), 30.0 % (w/w) ethanol, 2.5 % (w/w) phospholipid (1), 1.0 % (w/w) cholesterol (C), and 1.25 % (w/w) Tween 20 (z2T) or Tween 80 (z8T), prepared by stirring, 3 min homogenization (H) or 6 min homogenization (yH). Results are presented as mean value ± SD; $p < 0.0001$. Sample names explained in Table 6.

Sample	Directly after preparation		After 24 h in the refrigerator		After 14 Days in the refrigerator	
	Zeta Potential [mV]	± SD [mV]	Zeta Potential [mV]	± SD [mV]	Zeta Potential [mV]	± SD [mV]
C-yE-z8T-1	-30.00	0.20	-25.83	0.76	-39.17	0.21
H-C-yE-z8T-1	-25.70	0.95	-27.70	0.72	-28.90	0.26
yH-C-yE-z8T-1	-38.67	1.18	-31.90	2.69	-49.73	0.12
C-yE-z2T-1	-26.93	0.45	-29.37	0.35	-38.87	0.06
H-C-yE-z2T-1	-36.30	1.13	-27.17	1.53	-27.53	0.40
yH-C-yE-z2T-1	-27.93	0.75	-21.03	0.49	-24.80	0.35

Table S3. Zeta potential values of ethosomal formulations containing 10.0 % (w/w) ethylene glycol (yE), 30.0 % (w/w) ethanol, 2.5 % (w/w) phospholipid (1), 1.0 % (w/w) cholesterol (C) and 0.625 % (w/w) Tween 20 (x2T) or Tween 80 (x8T), prepared by 3 min homogenization (H) or 6 min homogenization (yH). Results are presented as mean value ± SD; $p < 0.0001$. Sample names explained in Table 6.

Sample	Directly after preparation		After 24 h in the refrigerator		After 14 days in the refrigerator	
	Zeta Potential [mV]	± SD [mV]	Zeta Potential [mV]	± SD [mV]	Zeta Potential [mV]	± SD [mV]
H-C-yE-x8T-1	-15.17	0.42	-25.70	0.72	-30.07	0.49
yH-C-yE-x8T-1	-31.57	2.06	-31.13	0.35	-30.83	0.47
H-C-yE-x2T-1	-37.40	0.75	-31.37	0.06	-31.33	0.15
yH-C-yE-x2T-1	-40.37	0.35	-34.03	0.31	-33.27	0.12

Table S4. Zeta potential values of ethosomal formulations prepared with surfactant mixtures of Tween 20/Mirasoft® SL L60 (xMixY) or Tween 80/Mirasoft® SL L60 (xMix). Formulations prepared with 10.0 % (w/w) ethylene glycol, 30.0 % (w/w) ethanol, 2.50 % (w/w) phospholipid, 1.0 % (w/w) cholesterol, and 0.625 % (w/w) total surfactant mixture, and by applying 3 min (H) or 6 min (yH) homogenization. Results are presented as mean value ± SD; $p < 0.009$. Sample names explained in Table 6.

Sample	Directly after preparation		After 24 h in the refrigerator		After 14 days in the refrigerator	
	Zeta Potential [mV]	± SD [mV]	Zeta Potential [mV]	± SD [mV]	Zeta Potential [mV]	± SD [mV]
H-C-yE-xMix-1	-50.87	2.36	-19.83	0.57	-28.40	0.17
yH-yE-xMix-1	-26.33	0.21	-24.2	0.40	-28.83	0.12
yH-C-yE-xMix-1	-22.07	0.42	-20.70	1.12	-24.17	0.25
H-C-yE-xMixY-1	-27.93	0.35	-31.67	0.74	-33.00	0.66
yH-yE-xMixY-1	-26.63	0.38	-44.63	0.31	-36.17	0.06
yH-C-yE-xMixY-1	-26.60	0.17	-25.50	0.44	-33.70	1.25

Table S5. Zeta potential values of ethosomal formulations prepared with reduced cholesterol content. Formulations prepared with 10.0 % (w/w) ethylene glycol, 30.0 % (w/w) ethanol, 2.5 % (w/w) phospholipid, 0.5 % (w/w) cholesterol (xC), and 0.625 % (w/w) total surfactant mixture: Tween 20/Mirasoft® SL L60 (xMixY) or Tween 80/Mirasoft® SL L60 (xMix), and by applying 3 min (H) or 6 min (yH) homogenization. Results are presented as mean value ± SD; p = 0.0002. Sample names explained in Table 6.

Sample	Directly after preparation		After 24 h in the refrigerator		After 14 days in the refrigerator	
	Zeta Potential [mV]	± SD [mV]	Zeta Potential [mV]	± SD [mV]	Zeta Potential [mV]	± SD [mV]
H-xC-yE-xMix-1	-22.63	0.32	-22.83	0.21	-29.07	0.32
yH-xC-yE-xMix-1	-20.73	0.31	-19.83	0.23	-23.33	0.38
H-xC-yE-xMixY-1	-21.47	0.65	-20.27	0.55	-21.43	0.15
yH-xC-yE-xMixY-1	-23.23	0.15	-21.57	0.67	-18.13	0.21

Table S6. Zeta potential values of betanin-loaded ethosomal formulations. Formulations prepared with 10.0 % (w/w) ethylene glycol, 30.0 % (w/w) ethanol, 2.50 % (w/w) phospholipid, 1.0 % (w/w) cholesterol, and 0.625 % (w/w) Tween 20/Mirasoft® SL L60 surfactant mixture (xMixY) or Tween 20 (x2T). Samples were loaded with 1.0, 3.0 or 5.0 % (w/w) betanin solution [7.5 mg/mL] and prepared by 3 min homogenization (H). Results presented as mean value ± SD; p < 0.0001. Sample names explained in Table 6.

Sample	Directly after preparation		After 24 h in the refrigerator		After 14 days in the refrigerator	
	Zeta Potential [mV]	± SD [mV]	Zeta Potential [mV]	± SD [mV]	Zeta Potential [mV]	± SD [mV]
H-C-yE-xMixY-1-xBe	-32.40	1.75	-26.77	2.47	-22.23	0.64
H-C-yE-xMixY-1-yBe	-34.27	1.42	-21.63	0.21	-24.13	0.15
H-C-yE-xMixY-1-zBe	-35.17	0.61	-27.03	0.25	-30.10	3.05
H-C-yE-x2T-1-xBe	-29.97	0.31	-28.00	0.53	-29.93	0.21
H-C-yE-x2T-1-yBe	-33.67	1.27	-28.20	0.82	-27.90	0.36
H-C-yE-x2T-1-zBe	-47.23	1.12	-29.90	0.17	-34.57	0.40

Table S7. General summary of the statistical analysis performed for physicochemical stability parameters.

**Measure**	**Value**
Number of statistical datasets	5
Total number of omnibus tests	123
one-way ANOVA tests	82
Kruskal-Wallis tests	41
Significant omnibus tests, p < 0.05	117
Non-significant omnibus tests	6
Significant omnibus tests [%]	95.1
Total number of post-hoc comparisons	729
Significant post-hoc comparisons, p < 0.05	406
Significant post-hoc comparisons [%]	55.7

Table S8. Summary of statistical results grouped according to the optimization stage.

**Optimization stage**	**Omnibus tests**	**Significant omnibus tests**	**Significant omnibus tests [%]**	**ANOVA**	**Kruskal-Wallis**	**Post-hoc comparisons**	**Significant post-hoc comparisons [%]**
Optimization of preparation method	27	25	92.6	17	10	183	50.3
Optimization of surfactant concentration	21	18	85.7	13	8	81	59.3
Optimization of surfactant type / biosurfactant addition	27	27	100.0	18	9	189	56.1
Optimization of cholesterol concentration	21	20	95.2	14	7	87	63.2
Betanin-loaded ethosomal formulations	27	27	100.0	20	7	189	55.6

Table S9. Summary of statistical results grouped by analyzed physicochemical parameter.

Parameter	Omnibus tests	Significant omnibus tests	Significant omnibus tests [%]	ANOVA	Kruskal-Wallis	Post-hoc comparisons	Significant post-hoc comparisons [%]
PDI	41	40	97.6	28	13	246	57.7
Zeta potential	41	39	95.1	26	15	243	46.5
Hydrodynamic diameter	41	38	92.7	28	13	240	62.9

Table S10. Mathematical equations employed for calculations of betanin release kinetics and their description

Name of equation	Equation	Legend	References
Zero-order	$Q_t = Q_0 + k_0 * t$	Q_t is the amount of drug dissolved in time t,<br>Q_0 is the initial amount of drug in the solution,<br>k_0 is the zero-order release constant	[93,118]]
First-order	$\log Q_t = \log Q_0 + k_1 * t$	k_1 is the first-order release constant	[93,119]
Higuchi	$Q_t = K_H * \sqrt{t}$	k_H is the Higuchi release constant	[93,120]
Hixson-Crowell	$Q_0^{1/3} – Q_t^{1/3} = k_{HC} * t$	k_{HC} is the Hixson-Crowell release constant	[93,121,122]
Korsmeyer-Peppas	$Q_t/Q_0 = k_{KP} * t^n$	k_{KP} is the Korsmeyer-Peppas release constant<br>n is the release generally used to analyze the release of pharmaceutical exponent, indicative of the drug release mechanism	[93,122–125]
Weibull	$\log [-\ln (1- (Q_t/Q_\infty))] = \beta * \log t – \beta \log \alpha$	α defines the time scale of the process,<br>β is shape parameter	[93,122,126]
RSS (Sum of square of residues)	$RSS = \sum_{i=1}^{N} (y_{i,exp} - y_{i,pred})^2$	$y_{i,exp}$ is measured value<br>$y_{i,pred}$ is predicted value	[116,117, 122,123,126]
AIC (Akaike Information Criterion)	$AIC = N * \ln (RRS/N) + 2 * P$	N is the number of measuring points,<br>P is the number of model's parameters	[117, 122,125,126]
AICc (Corrected Akaike Information Criterion)	$AICc = AIC + \frac{2*(P*(P+1))}{(N-P-1)}$		[116,117, 122,123,126]

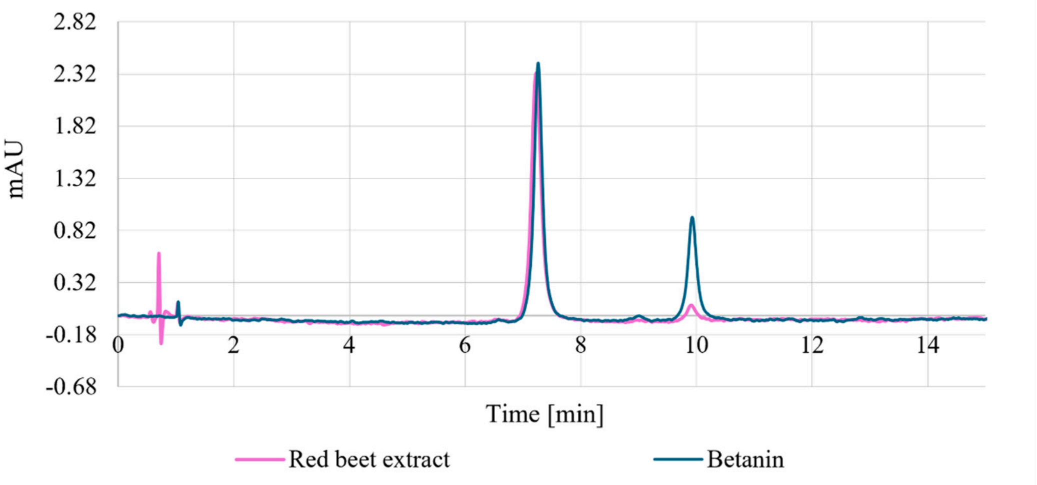

Figure S1. Chromatogram HPLC of red beet extract and betanin solution.

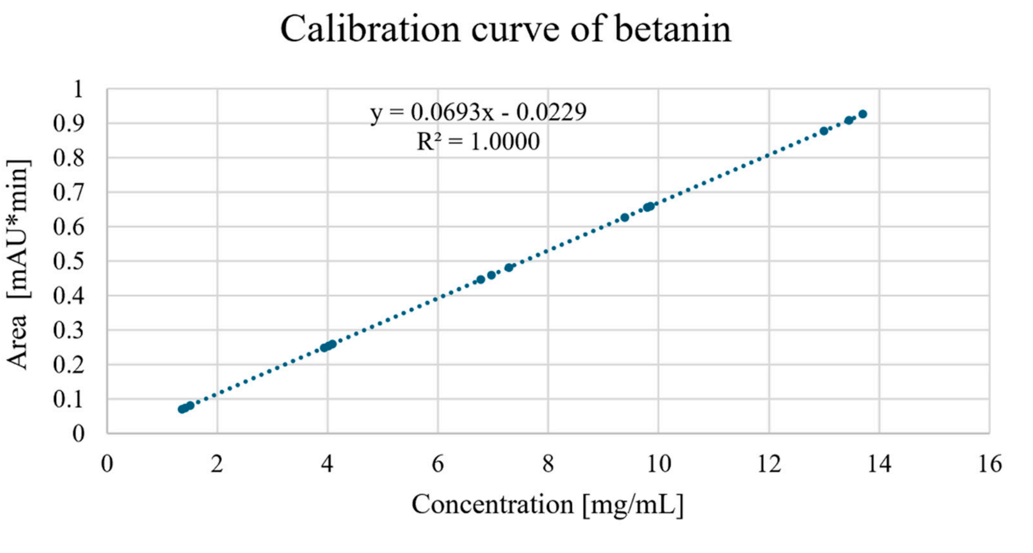

Figure S2. Calibration curve of betanin solution in water.

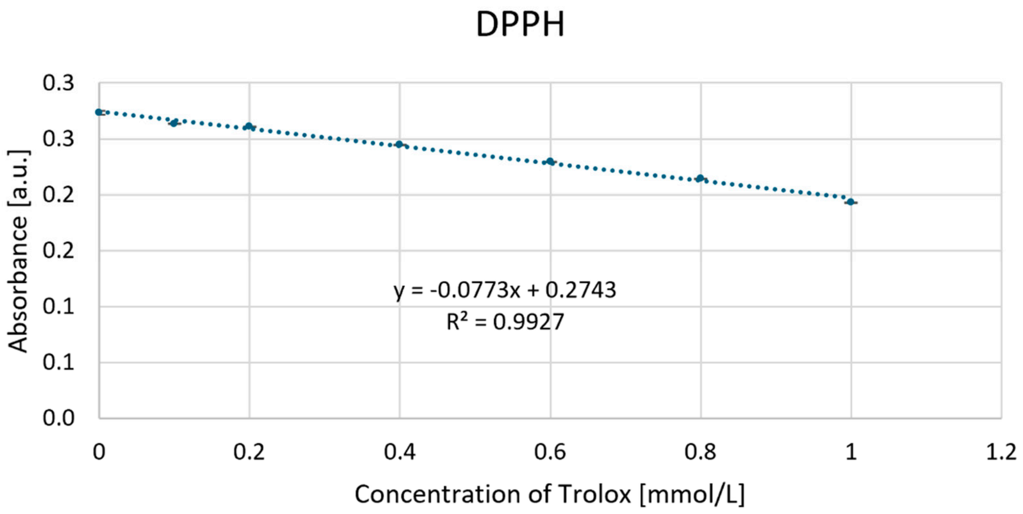

Figure S3. Calibration curve of DPPH assay.

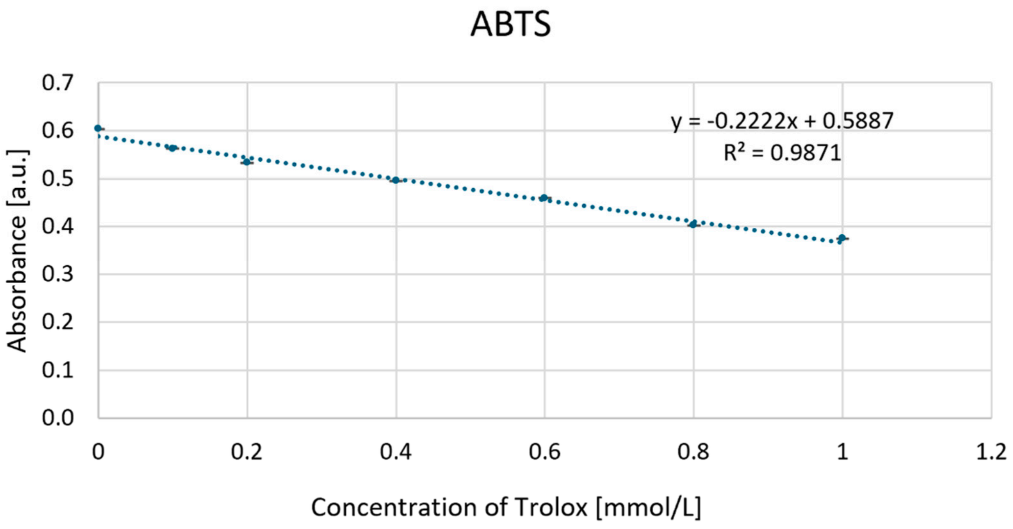

Figure S4. Calibration curve of ABTS assay.

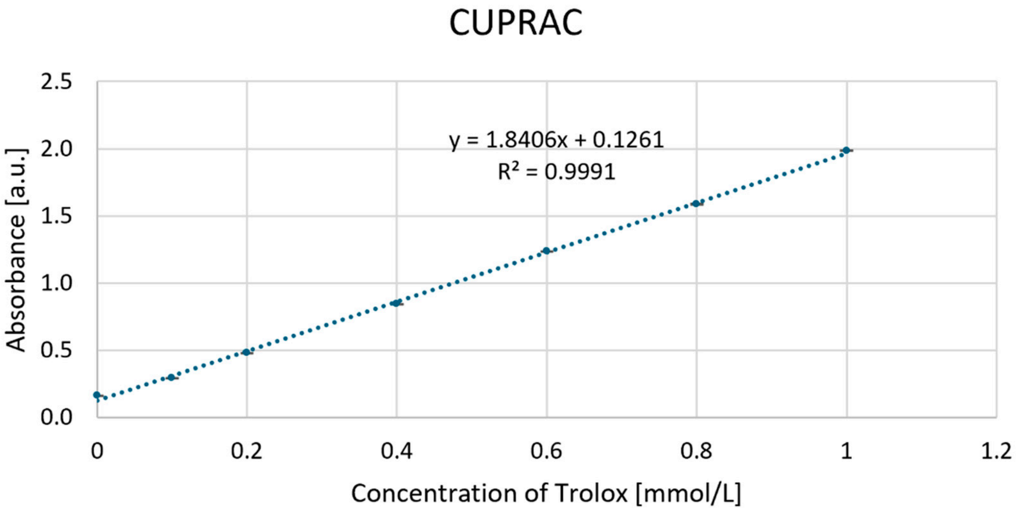

Figure S5. Calibration curve of CUPRAC assay.

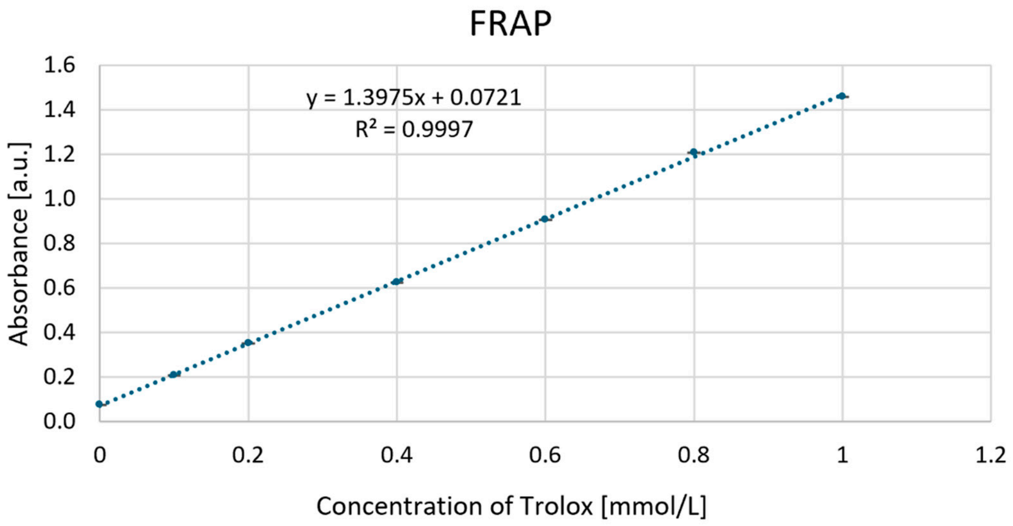

Figure S6. Calibration curve of FRAP assay.

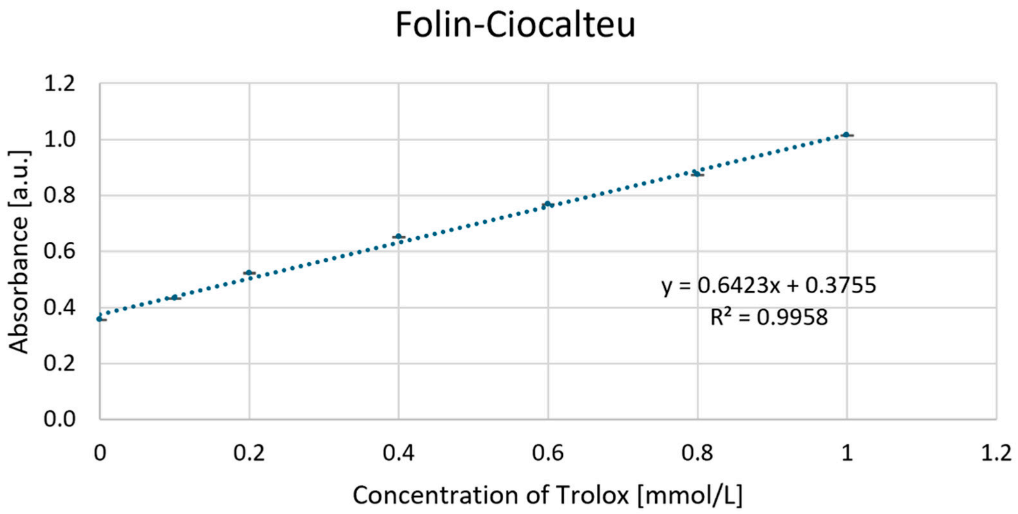

Figure S7. Calibration curve of Folin-Ciocalteu assay.

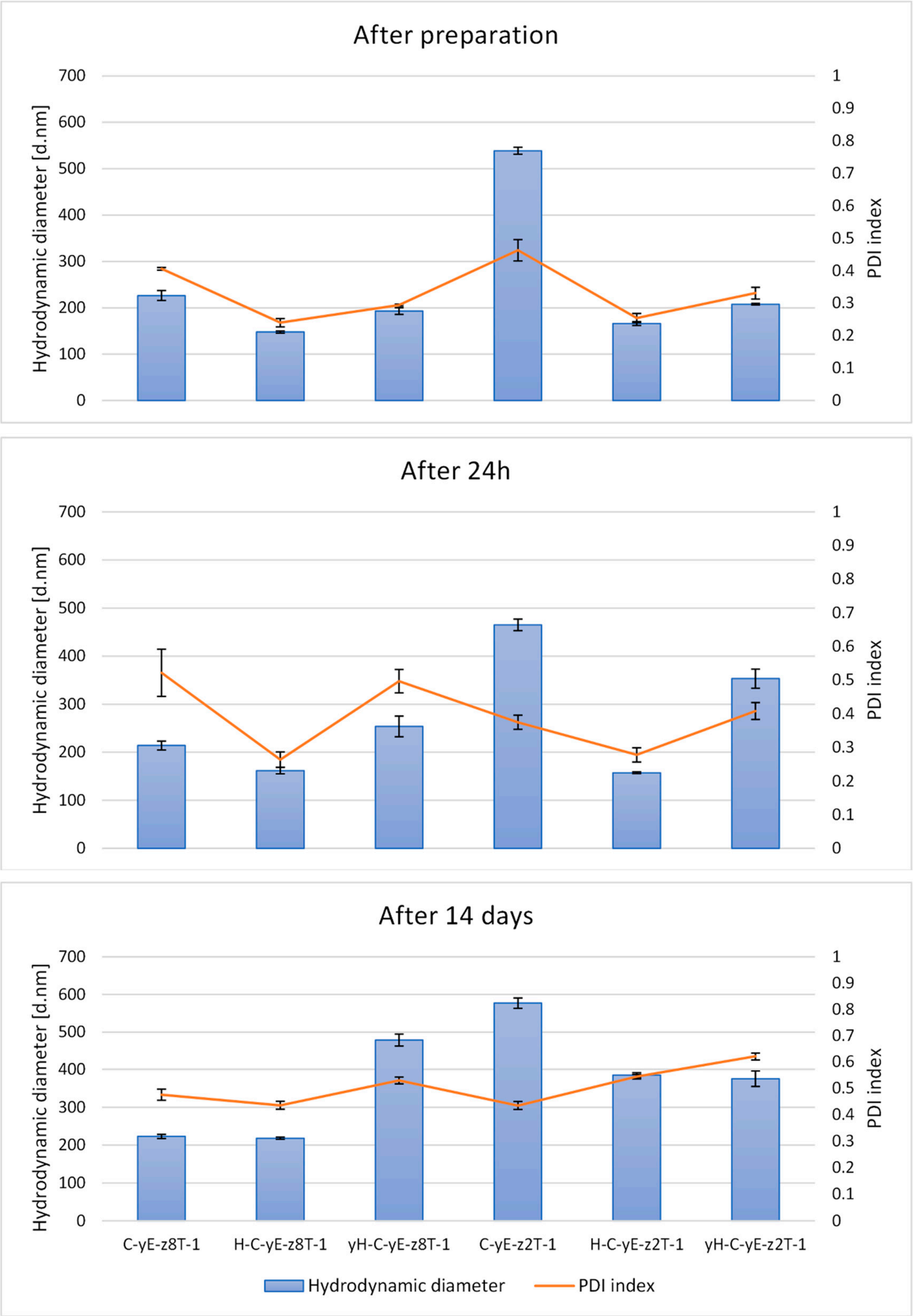

Figure S8. D_h and PDI of ethosomal formulations containing 10.0 % (w/w) ethylene glycol (yE), 30.0 % (w/w) ethanol, 2.5 % (w/w) phospholipid (1), 1.0 % (w/w) cholesterol (C) and 1.25 % (w/w) Tween 20 (z2T) or Tween 80 (z8T), prepared by stirring, 3 min homogenization (H) or 6 min homogenization (yH). D_h: $p < 0.005$; PDI: $p = 0.006$. Sample names explained in Table 6.

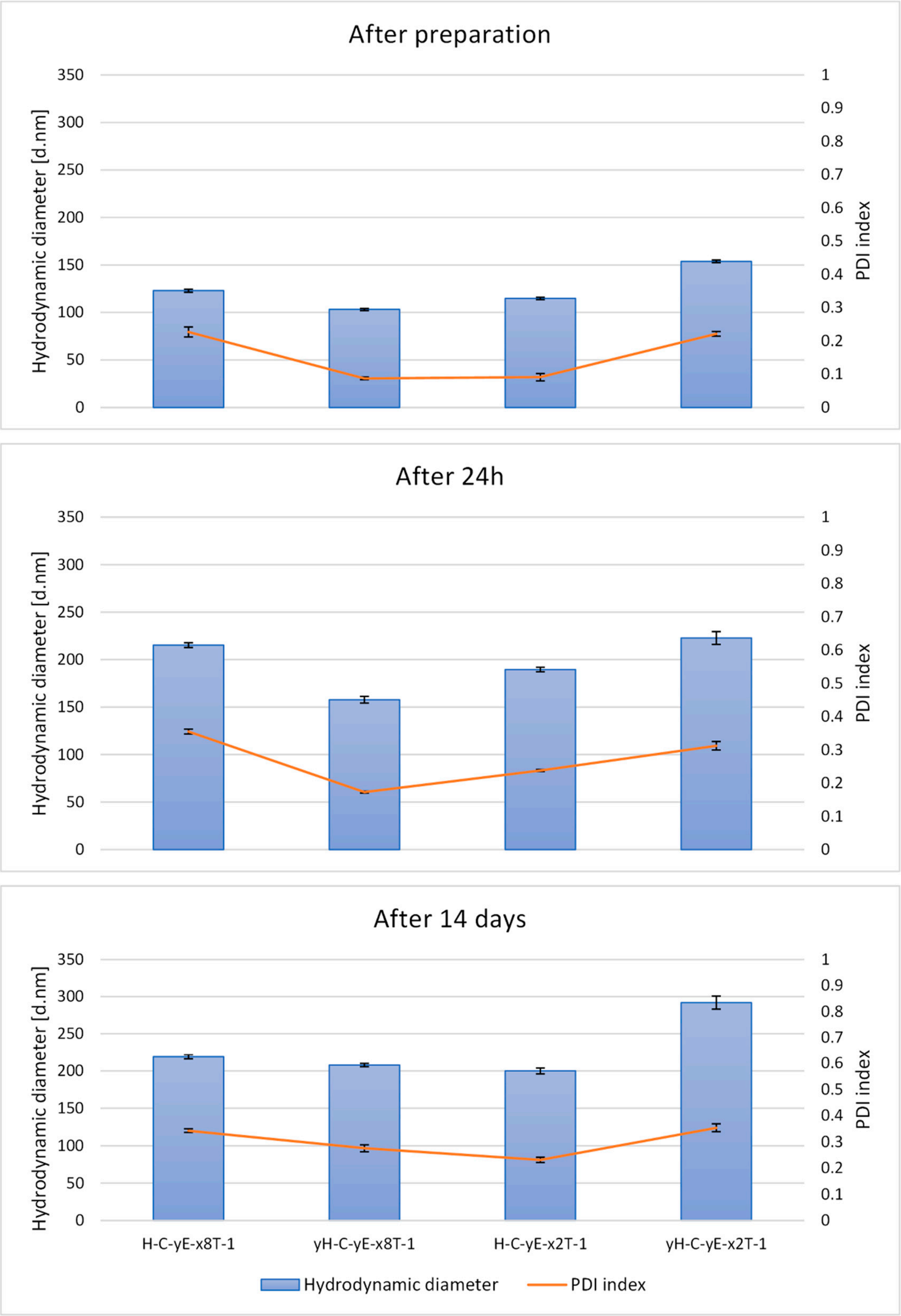

Figure S9. D_h and PDI of ethosomal formulations containing 10.0 % (w/w) ethylene glycol (yE), 30.0 % (w/w) ethanol, 2.5 % (w/w) phospholipid (1), 1.0 % (w/w) cholesterol (C) and 0.625 % (w/w) Tween 20 (x2T) or Tween 80 (x8T), prepared by 3 min homogenization (H) or 6 min homogenization (yH). D_h: $p < 0.0001$; PDI: $p < 0.0001$. Sample names explained in Table 6.

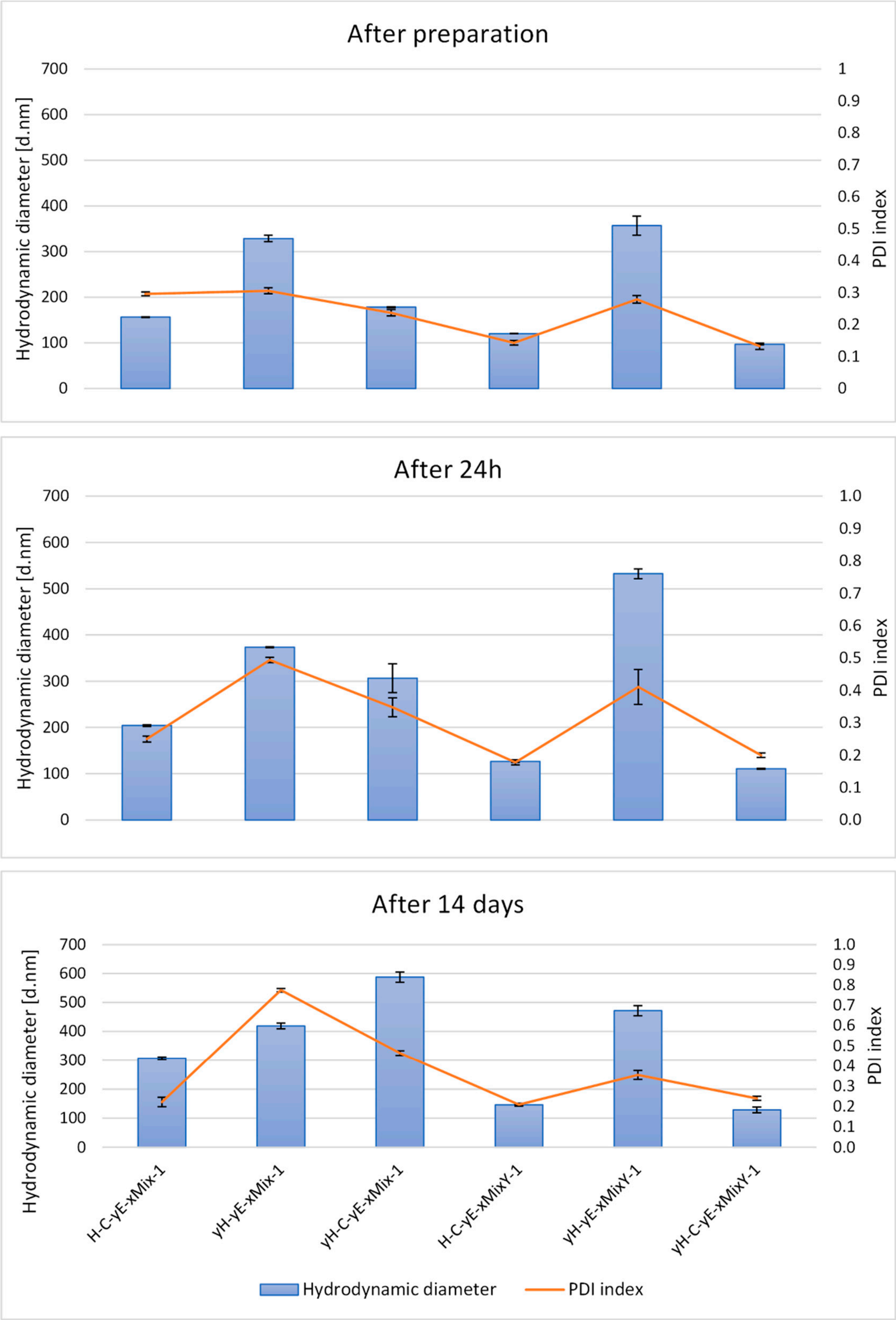

Figure S10. D_h and PDI of ethosomal formulations prepared with surfactant mixtures of Tween 20/Mirasoft® SL L60 (xMixY) or Tween 80/Mirasoft® SL L60 (xMix). Formulations prepared with 10 % (w/w) ethylene glycol, 30 % (w/w) ethanol, 2.5 % (w/w) phospholipid, 1 % (w/w) cholesterol and 0.625 % (w/w) total surfactant mixture, and by applying 3 min (H) or 6 min (yH) homogenization. D_h: $p < 0.005372$; PDI: $p < 0.0001$. Sample names explained in Table 6.

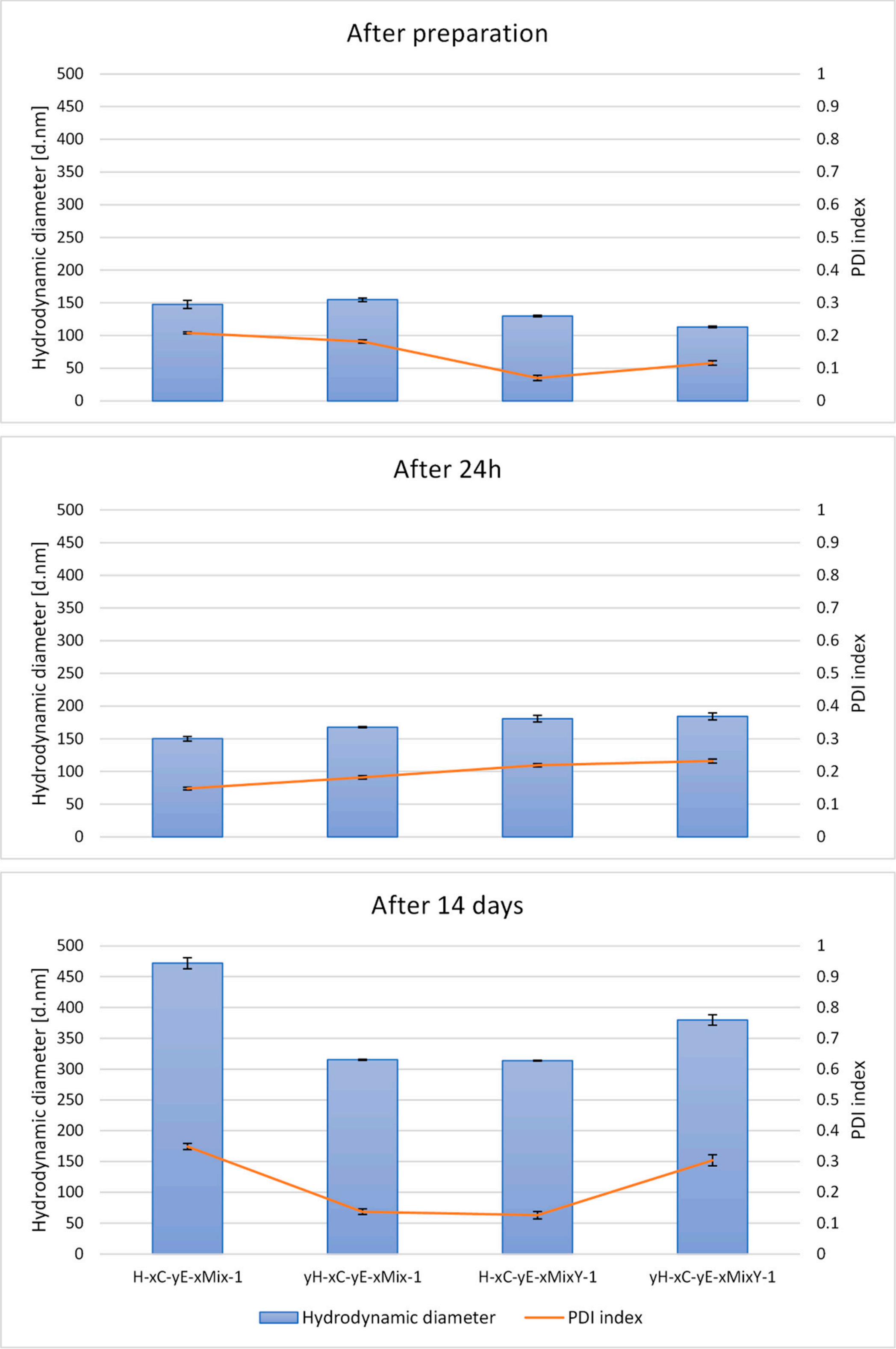

Figure S11. D_h and PDI of ethosomal formulations prepared with reduced cholesterol content. Formulations prepared with 10.0 % (w/w) ethylene glycol, 30.0 % (w/w) ethanol, 2.50 % (w/w) phospholipid, 0.50 % (w/w) cholesterol (xC), and 0.625 % (w/w) total surfactant mixture: Tween 20/Mirasoft® SL L60 (xMixY) or Tween 80/Mirasoft® SL L60 (xMix), and by applying 3 min (H) or 6 min (yH) homogenization. D_h: $p = 0.0216$; PDI: $p < 0.0001$. Sample names explained in Table 6.

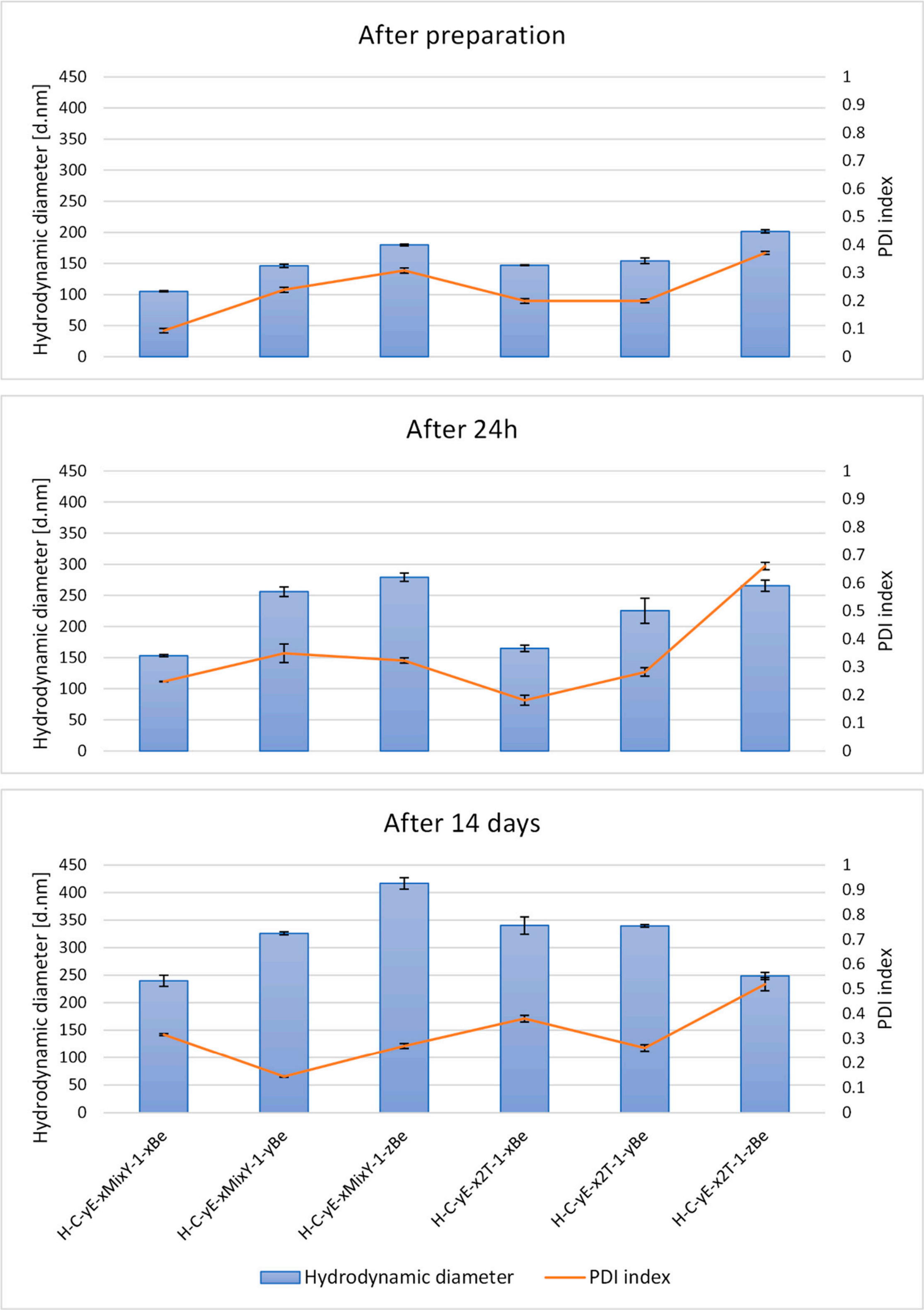

Figure S12. D_h and PDI of betanin-loaded ethosomal formulations. Formulations prepared with 10.0 % (w/w) ethylene glycol, 30.0 % (w/w) ethanol, 2.5 % (w/w) phospholipid, 1.0 % (w/w) cholesterol, and 0.625 % (w/w) Tween 20/Mirasoft® SL L60 surfactant mixture (xMixY) or Tween 20 (x2T); Loaded with 1.0, 3.0 or 5.0 % (w/w) betanin solution [7.5 mg/mL] and prepared by 3 min homogenization (H). D_h: $p < 0.0001$; PDI: $p < 0.0001$. Sample names explained in Table 6.

a)

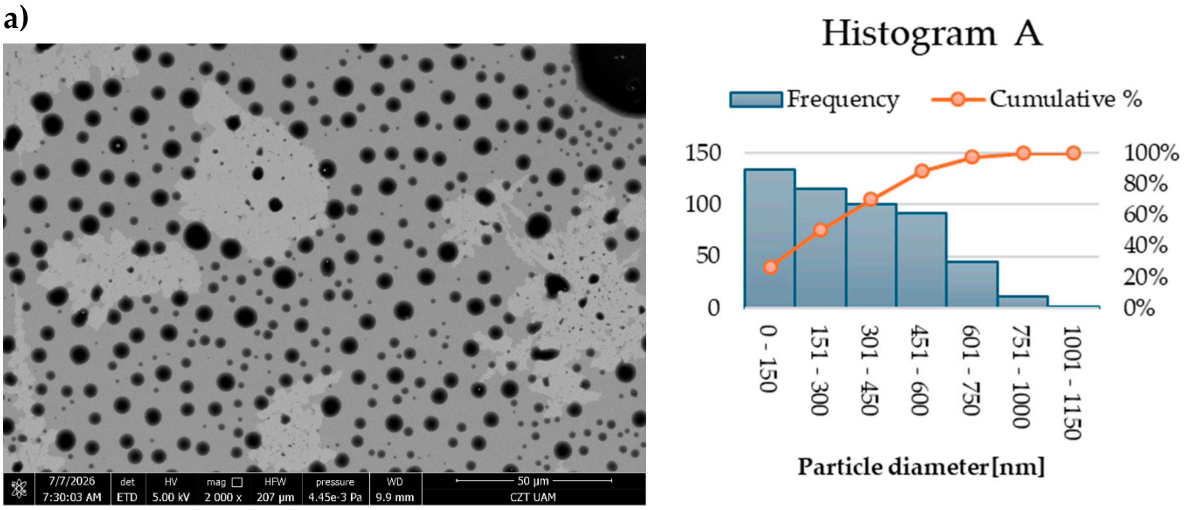

b)

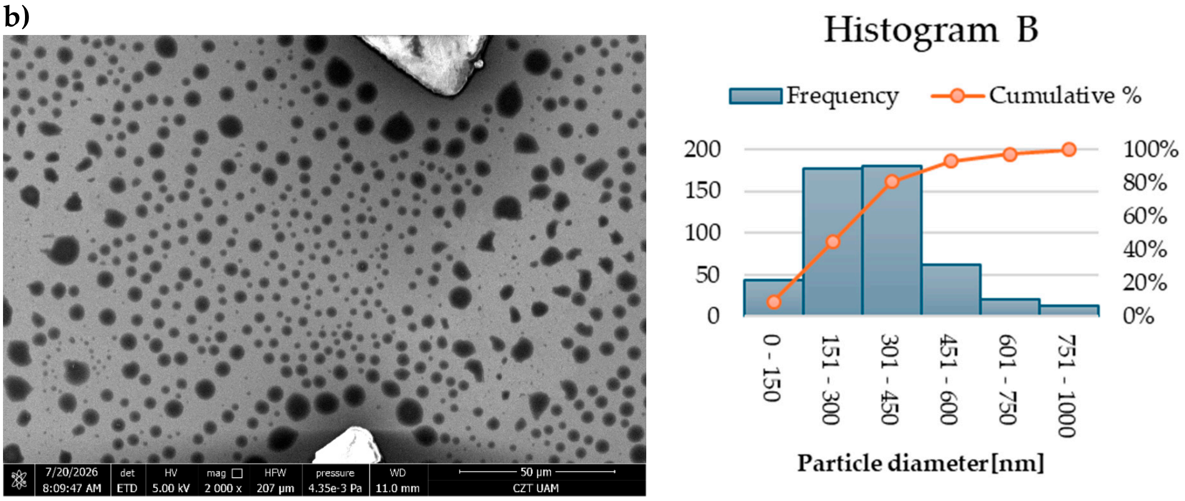

Figure S13. SEM image after a) 24 h; b) 14 days in the refrigerator (accelerating voltage of 5 kV) showing ethosomes in a sample based on 10.0 % (w/w) of ethylene glycol (yE), 30.0 % (w/w) of ethanol and 2.5 % (w/w) of phospholipid (1), 1.0 % (w/w) of cholesterol (C), 0.625 (x) % (w/w) mixture of surfactants: Mirasoft® SL L60 and Tween 20 (MixY) and 1.0 % (w/w) of betanin solution (xBe). Magnification used was: 2,000 times (names of the samples according to Table 6).

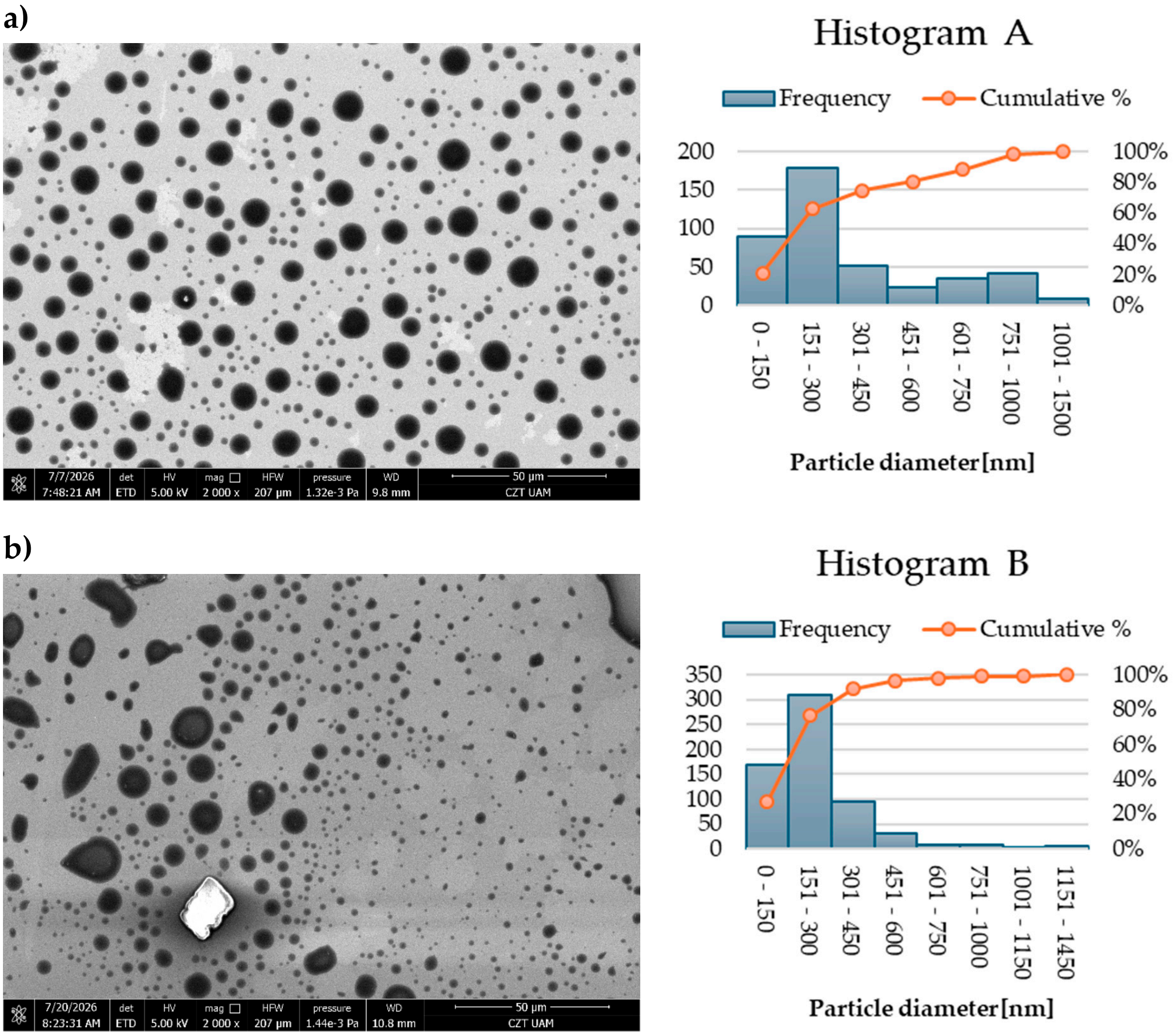

Figure S14. SEM image after a) 24 h; b) 14 days in the refrigerator (accelerating voltage of 5 kV) showing ethosomes in a sample based on 10.0 % (w/w) of ethylene glycol (yE), 30.0 % (w/w) of ethanol and 2.5 % (w/w) of phospholipid (1), 1.0 % (w/w) of cholesterol (C), 0.625 (x) % (w/w) mixture of surfactants: Mirasoft® SL L60 and Tween 20 (MixY) and 3.0 % (w/w) of betanin solution (yBe). Magnification used was: 2,000 times (names of the samples according to Table 6).

a)

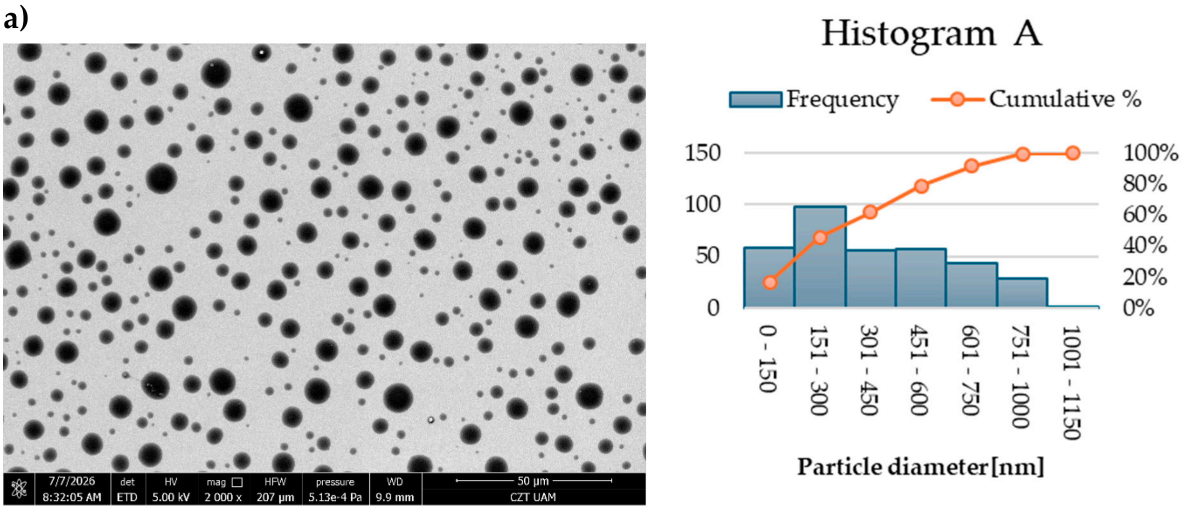

b)

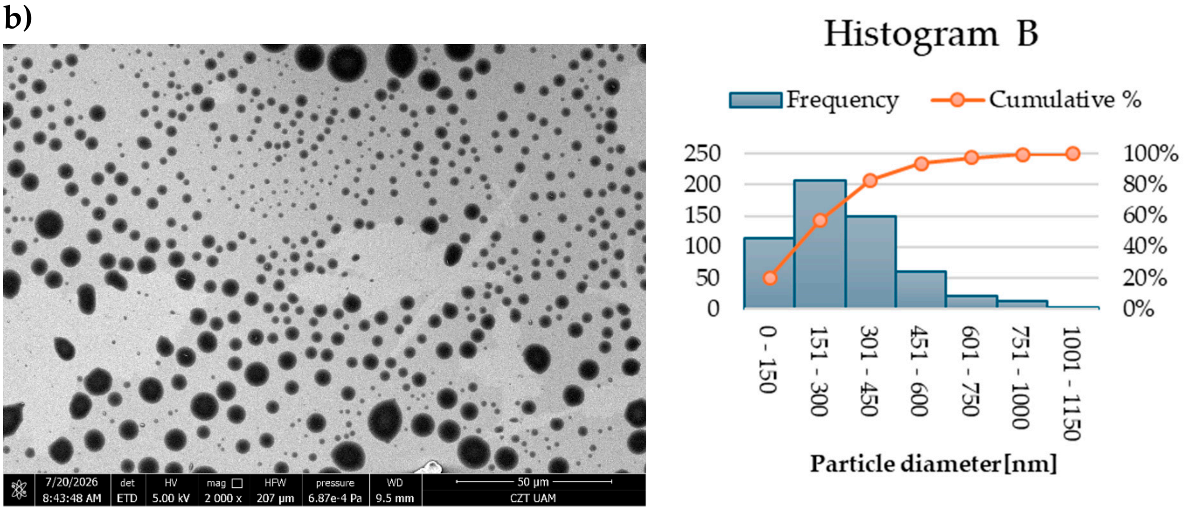

Figure S15. SEM image after a) 24 h; b) 14 days in the refrigerator (accelerating voltage of 5 kV) showing ethosomes in a sample based on 10.0 % (w/w) of ethylene glycol (yE), 30.0 % (w/w) of ethanol and 2.5 % (w/w) of phospholipid (1), 1.0 % (w/w) of cholesterol (C), 0.625 % (w/w) (x) mixture of surfactants: Mirasoft® SL L60 and Tween 20 (MixY) and 5.0 % (w/w) of betanin solution (zBe). Magnification used was: 2,000 times (names of the samples according to Table 6).

a)

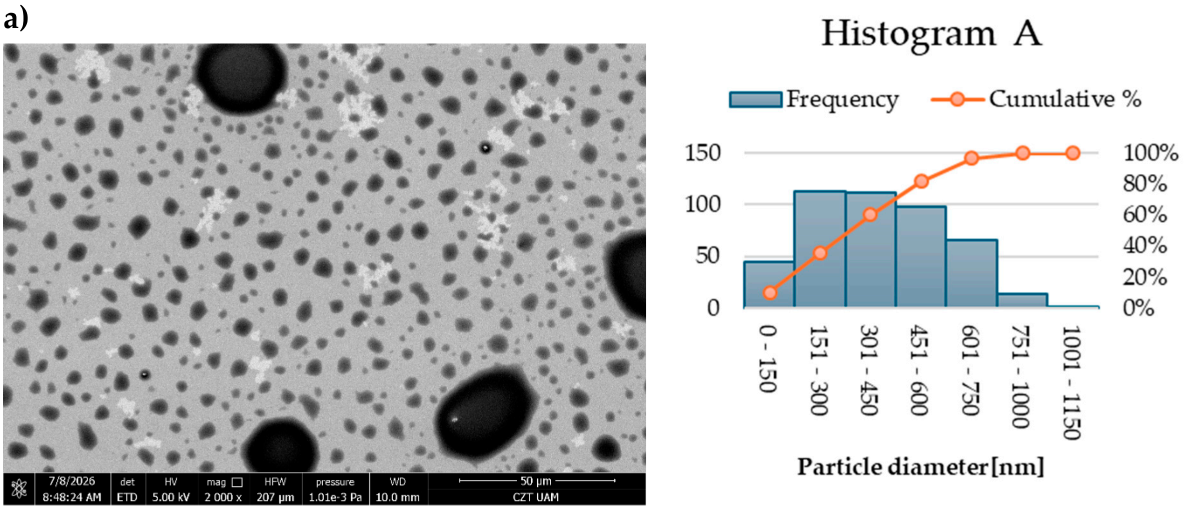

b)

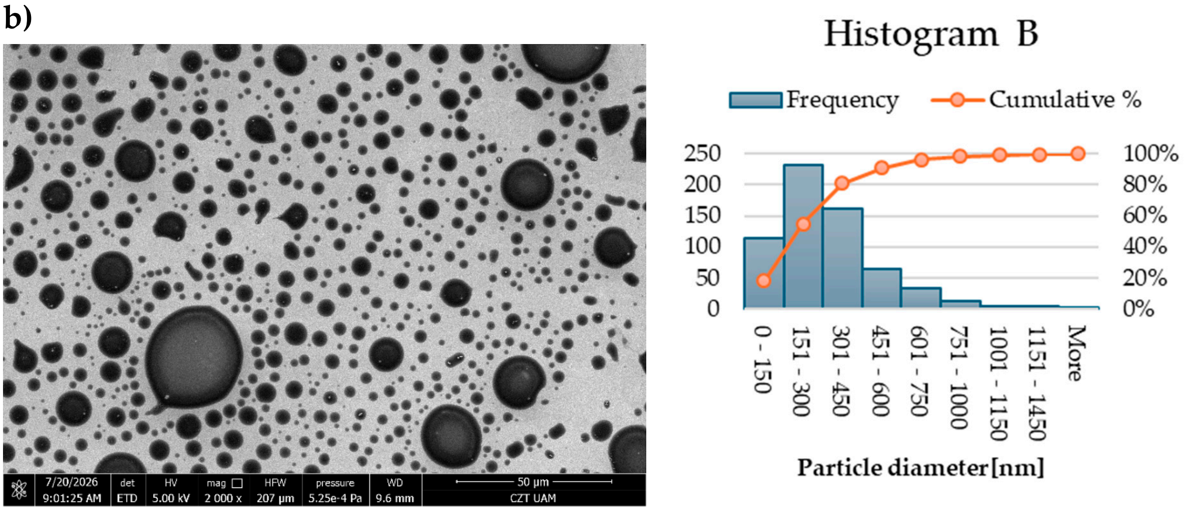

Figure S16. SEM image after a) 24 h; b) 14 days in the refrigerator (accelerating voltage of 5 kV) showing ethosomes in a sample based on 10.0 % (w/w) of ethylene glycol (yE), 30.0 % (w/w) of ethanol and 2.5 % (w/w) of phospholipid (1), 1.0 % (w/w) of cholesterol (C), 0.625 % (w/w) (x) of surfactants: Mirasoft® SL L60 and Tween 20 (MixY). Magnification used was: 2,000 times (names of the samples according to Table 6).

a)

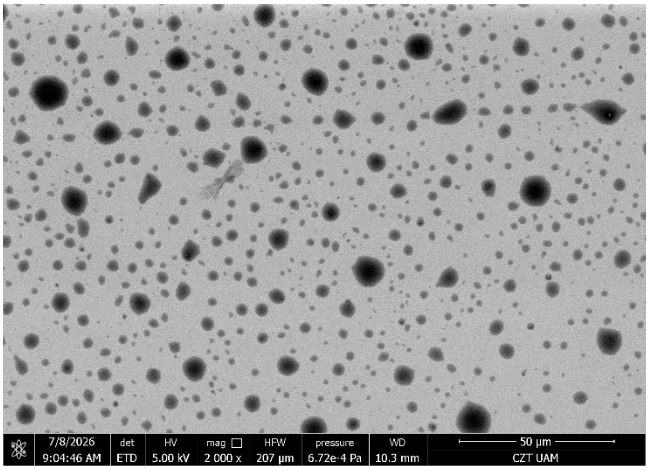

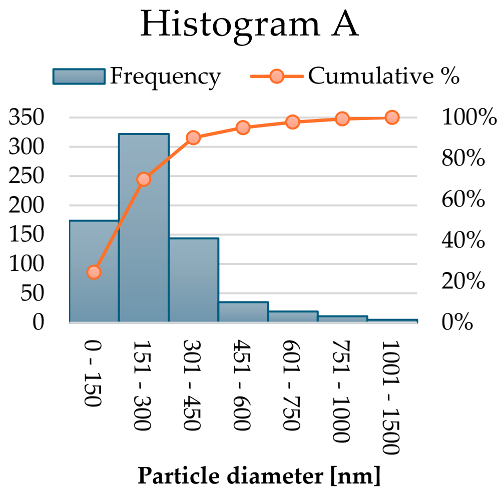

b)

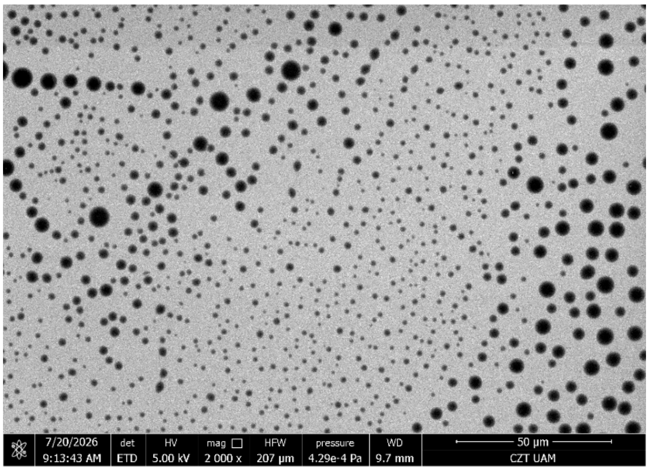

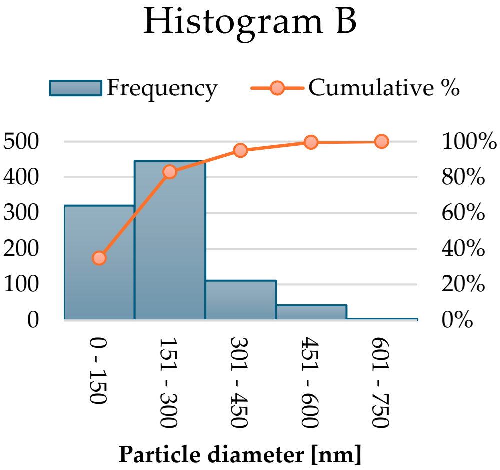

Figure S17. SEM image after a) 24 h; b) 14 days in the refrigerator (accelerating voltage of 5 kV) showing ethosomes in a sample based on 10.0 % (w/w) of ethylene glycol (yE), 30.0 % (w/w) of ethanol and 2.5 % (w/w) of phospholipid (1), 1.0 % (w/w) of cholesterol (C), 0.625 % (w/w) (x) of surfactants: Tween 20 (2T) and 1.0 % (w/w) of betanin solution (xBe). Magnification used was: 2,000 times (names of the samples according to Table 6).

a)

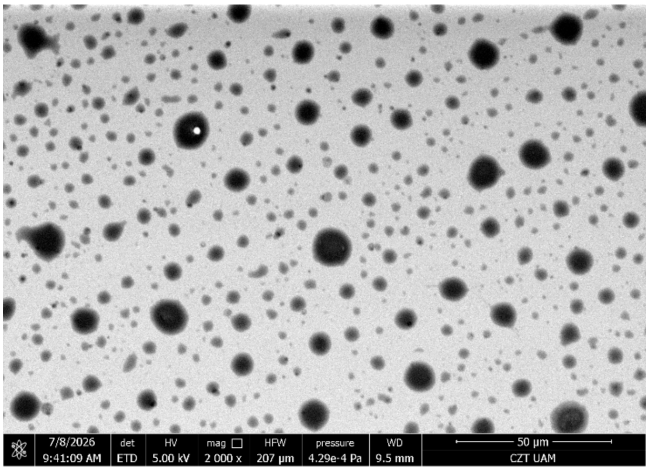

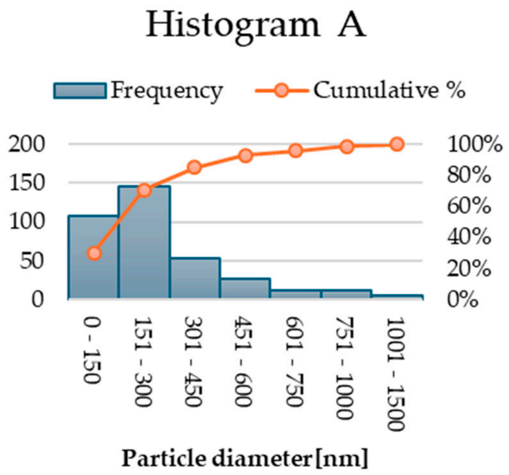

b)

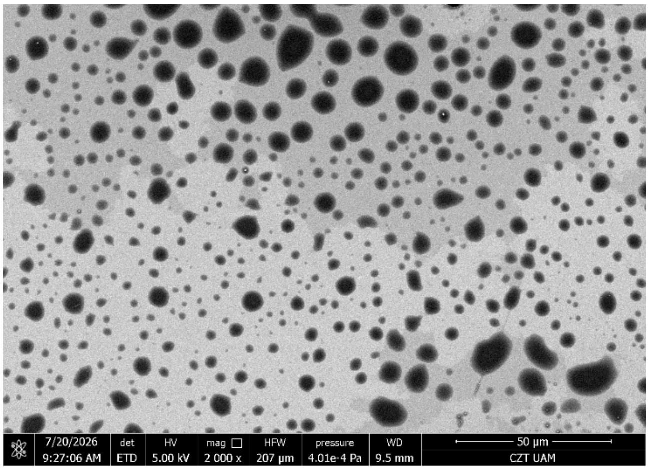

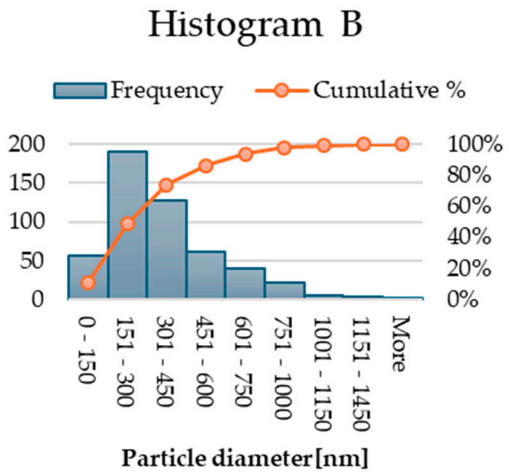

Figure S18. SEM image after a) 24 h; b) 14 days in the refrigerator (accelerating voltage of 5 kV) showing ethosomes in a sample based on 10 % (w/w) of ethylene glycol (yE), 30.0 % (w/w) of ethanol and 2.5 % (w/w) of phospholipid (1), 1.0 % (w/w) of cholesterol (C), 0.625 % (w/w) (x) of surfactants: Tween 20 (2T) and 3.0 % (w/w) of betanin solution (yBe). Magnification used was: 2,000 times (names of the samples according to Table 6).

a)

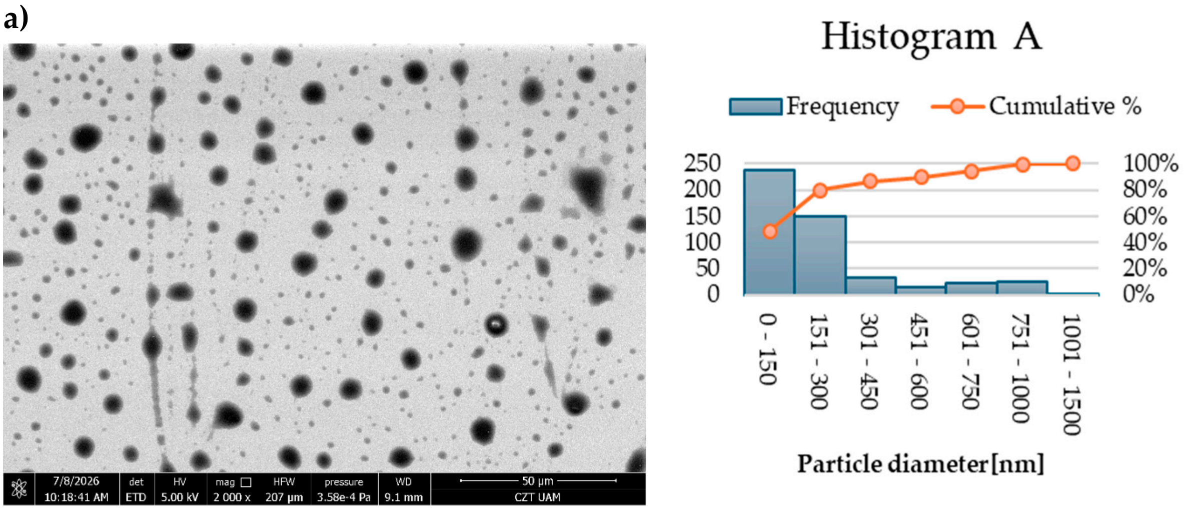

b)

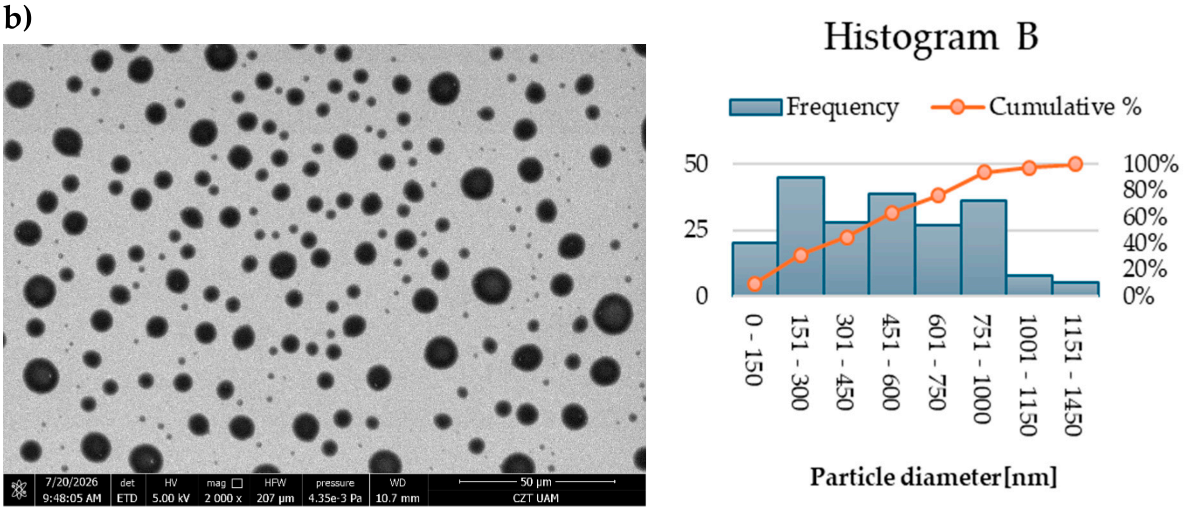

Figure S19. SEM image after a) 24 h; b) 14 days in the refrigerator (accelerating voltage of 5 kV) showing ethosomes in a sample based on 10.0 % (w/w) of ethylene glycol (yE), 30.0 % (w/w) of ethanol and 2.5 % (w/w) of phospholipid (1), 1.0 % (w/w) of cholesterol (C), 0.625 % (w/w) (x) of surfactants: Tween 20 (2T) and 5.0 % (w/w) of betanin solution (zBe). Magnification used was: 2,000 times (names of the samples according to Table 6).

a)

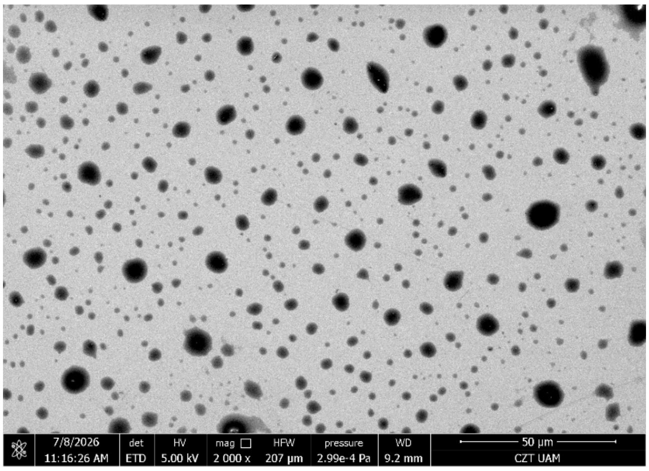

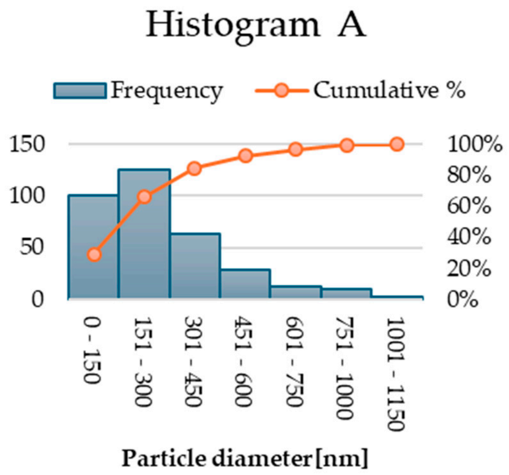

b)

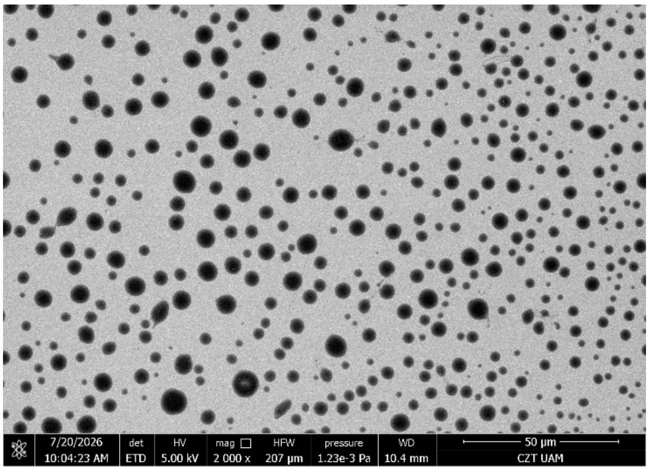

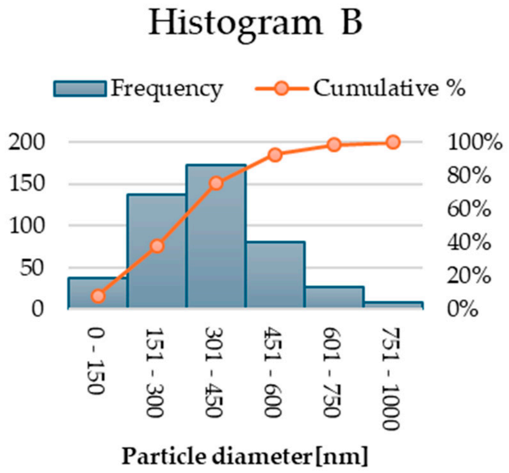

Figure S20. SEM image after a) 24 h; b) 14 days in the refrigerator (accelerating voltage of 5 kV) showing ethosomes in a sample based on 10.0 % (w/w) of ethylene glycol (yE), 30.0 % (w/w) of ethanol and 2.5 % (w/w) of phospholipid (1), 1.0 % (w/w) of cholesterol (C), 0.625 % (w/w) (x) of surfactants: Tween 20 (2T). Magnification used was: 2,000 times (names of the samples according to Table 6).
